# Research needs in allergy: an EAACI position paper, in collaboration with EFA

**DOI:** 10.1186/2045-7022-2-21

**Published:** 2012-11-02

**Authors:** Nikolaos G Papadopoulos, Ioana Agache, Sevim Bavbek, Beatrice M Bilo, Fulvio Braido, Victoria Cardona, Adnan Custovic, Jan deMonchy, Pascal Demoly, Philippe Eigenmann, Jacques Gayraud, Clive Grattan, Enrico Heffler, Peter W Hellings, Marek Jutel, Edward Knol, Jan Lötvall, Antonella Muraro, Lars K Poulsen, Graham Roberts, Peter Schmid-Grendelmeier, Chrysanthi Skevaki, Massimo Triggiani, Ronald vanRee, Thomas Werfel, Breda Flood, Susanna Palkonen, Roberta Savli, Pia Allegri, Isabella Annesi-Maesano, Francesco Annunziato, Dario Antolin-Amerigo, Christian Apfelbacher, Miguel Blanca, Ewa Bogacka, Patrizia Bonadonna, Matteo Bonini, Onur Boyman, Knut Brockow, Peter Burney, Jeroen Buters, Indre Butiene, Moises Calderon, Lars Olaf Cardell, Jean-Christoph Caubet, Sevcan Celenk, Ewa Cichocka-Jarosz, Cemal Cingi, Mariana Couto, Nicolette deJong, Stefano Del Giacco, Nikolaos Douladiris, Filippo Fassio, Jean-Luc Fauquert, Javier Fernandez, Montserrat Fernandez Rivas, Marta Ferrer, Carsten Flohr, James Gardner, Jon Genuneit, Philippe Gevaert, Anna Groblewska, Eckard Hamelmann, Hans Jürgen Hoffmann, Karin Hoffmann-Sommergruber, Lilit Hovhannisyan, Valérie Hox, Frode L Jahnsen, Ömer Kalayci, Ayse Füsun Kalpaklioglu, Jörg Kleine-Tebbe, George Konstantinou, Marcin Kurowski, Susanne Lau, Roger Lauener, Antti Lauerma, Kirsty Logan, Antoine Magnan, Joanna Makowska, Heidi Makrinioti, Paraskevi Mangina, Felicia Manole, Adriano Mari, Angel Mazon, Clare Mills, ErvinÇ Mingomataj, Bodo Niggemann, Gunnar Nilsson, Markus Ollert, Liam O'Mahony, Serena O'Neil, Gianni Pala, Alberto Papi, Gianni Passalacqua, Michael Perkin, Oliver Pfaar, Constantinos Pitsios, Santiago Quirce, Ulrike Raap, Monika Raulf-Heimsoth, Claudio Rhyner, Paula Robson-Ansley, Rodrigo Rodrigues Alves, Zeljka Roje, Carmen Rondon, Odilija Rudzeviciene, Franziska Ruëff, Maia Rukhadze, Gabriele Rumi, Cansin Sackesen, Alexandra F Santos, Annalisa Santucci, Christian Scharf, Carsten Schmidt-Weber, Benno Schnyder, Jürgen Schwarze, Gianenrico Senna, Svetlana Sergejeva, Sven Seys, Andrea Siracusa, Isabel Skypala, Milena Sokolowska, Francois Spertini, Radoslaw Spiewak, Aline Sprikkelman, Gunter Sturm, Ines Swoboda, Ingrid Terreehorst, Elina Toskala, Claudia Traidl-Hoffmann, Carina Venter, Berber Vlieg-Boerstra, Paul Whitacker, Margitta Worm, Paraskevi Xepapadaki, Cezmi A Akdis

**Affiliations:** 1 Allergy Department, 2nd Pediatric Clinic, University of Athens, Athens, Greece; 2 Transylvania University, Brasov, Romania; 3 Ankara University, Ankara, Turkey; 4 University Hospital Ospedali Riuniti, Ancona, Italy; 5 Allergy & Respiratory Diseases Clinic – DIMI, University of Genoa, IRCCS AOU San Martino-IST, Genoa, Italy; 6 Hospital Vall d'Hebron, Barcelona, Spain; 7 University of Manchester, Manchester, United Kingdom; 8 University Hospital Groningen, Groningen, The Netherlands; 9 University Hospital of Montpellier, Montpellier, France; 10 Children's Hospital, Geneva, Switzerland; 11 Polyclinique de l’Ormeau, Tarbes, France; 12 Norfolk & Norwich University Hospital, Norwich, United Kingdom; 13 Dipartimento di Scienze Mediche, University of Torino, Torino, Italy; 14 University Hospitals Leuven, Leuven, Belgium; 15 Wroclaw Medical University, Wroclaw, Poland; 16 University Medical Center Utrecht, Utrecht, The Netherlands; 17 Krefting Research Centre, Gothenburg, Sweden; 18 Department of Pediatrics, University of Padua, Padova, Italy; 19 Copenhagen University Hospital at Gentofte, Gentofte, Denmark; 20 University of Southampton Faculty of Medicine, Southampton, United Kingdom; 21 University of Zurich, Zurich, Switzerland; 22 University of Salerno, Fisciano, Salerno, Italy; 23 Academic Medical Center, Amsterdam, The Netherlands; 24 Department of Dermatology and Allergy, Hannover Medical School, Hannover, Germany; 25 European Federation of Allergy and Airways Diseases Patients‘ Associations (EFA), Brussels, Belgium; 26 Uveitis Center, Ophthamology epmn, Rapallo Hospital, Genova, Italy; 27 Epidemiology of Allergic and Respiratory Disease, UMR-S 707 INSERM and UPMC Paris Sorbonnes, Paris, France; 28 University of Florence, Florence, Italy; 29 Hospital Universitario Príncipe de Asturias, Madrid, Spain; 30 University of Regensburg, Germany / Brighton and Sussex Medical School, Brighton, United Kingdom; 31 Research Laboratory, Carlos Hava Hospital, Malaga, Spain; 32 Department of Internal Diseases, Geriatrics and Allergology, University of Medicine, Wroclaw, Poland; 33 Allergy Unit, Azienda Ospedaliera Universitaria Integrata, Padova, Italy; 34 Department of Internal Medicine, Lung Function Unit, "Sapienza", University of Rome, Rome, Italy; 35 Department of Dermatology, Technische Universität München, Munich, Germany; 36 Imperial College London, London, United Kingdom; 37 CK-CARE, ZAUM – Center of Allergy & Environment, Helmholtz Zentrum München/Technische Universität, Munich, Munich, Germany; 38 Vilnius University, Vilnius, Lithuania; 39 Royal Brompton Hospital, London, United Kingdom; 40 Karolinska University Hospital, Stockholm, Sweden; 41 University Hospitals of Geneva, Medical School of the University of Geneva, Department of Child and Adolescent Medicine, Geneva, Switzerland; 42 Uludag University, Bursa, Turkey; 43 Chair and Department of Pediatrics, Jagiellonian University Medical College, Krakow, Poland; 44 Department of Otorhinolaryngology, Head and Neck Surgery, Eskisehir Osmangazi University, Eskisehir, Turkey; 45 Centro Hospitalar São João EPE, Porto, Portugal; 46 ErasmusMC, Rotterdam, The Netherlands; 47 Department of Medical Sciences “M. Aresu”, University of Cagliari, Cagliari, Italy; 48 Careggi Hospital, Florence, Italy; 49 Pédiatre A, CHRU Clermont Ferrand, Clermont Ferrand, France; 50 UMH University, Alicante, Spain; 51 Hospital Clínico San Carlos, Madrid, Spain; 52 Universidad de Navarra, Pamplona, Spain; 53 St Thomas' Hospital & King's College London, London, UK; 54 Royal Free Hospital, London, United Kingdom; 55 Institute of Epidemiology and Medical Biometry, Ulm University, Ulm, Germany; 56 University Hospital Ghent, Ghent, Belgium; 57 Polish Mother’s Memorial Hospital - Research Institute, Department of Opthalmology, Lodz, Poland; 58 Klinik für Kinder und Jugendmedizin, St. Josef Hospital Ruhr University, Bochum, Germany; 59 Aarhus University Hospital, Aarhus, Denmark; 60 Medical University Vienna, Wien, Austria; 61 Institute of Molecular Biology, Yerevan, Armenia; 62 Oslo University Hospital, Rikshospitalet, Oslo, Norway; 63 Pediatric Allergy and Asthma Unit, Ihsan Dogramaci Children’s Hospital, Hacettepe Univirsity School of Medicine, Ankara, Turkey; 64 Allergie- & Asthma-Zentrum Berlin Westend, Berlin, Germany; 65 Department of Allergy and Clinical Immunology, 424 General Military Training Hospital, Thessaloniki, Greece; 66 Department of Immunology, Rheumatology and Allergy, Medical University of Lodz, Lodz, Poland; 67 Charité C. Virchow University Children`s Hospital, Berlin, Germany; 68 Hochgebirgsklinik, Davos-Wolfgang, Davos, Switzerland; 69 Skin and Allergy Hospital, Helsinki, Finland; 70 King's College London, London, United Kingdom; 71 L'Institut du Thorax, Nantes, France; 72 Department of Allergy and Clinical Immunology, Lodz, Poland; 73 Faculty of Medicine, ENT Department, University of Oradea, Oradea, Romania; 74 Center for Molecular Allergology, IDI-IRCCS, Rome, Italy; 75 Unit of Pediatric allergy and Pneumology, Children’s Hospital La Fe, Valencia, Spain; 76 Manchester Interdisciplinary Biocentre, Manchester, United Kingdom; 77 Department of Allergology and Clinical Immunology, Mother Theresa School of Medicine, Tirana, Albania; 78 German Red Cross Hospital Westend, Berlin, Germany; 79 Centre for Allergy Research at Karolinska Institutet, Stockholm, Sweden; 80 Technical University of Munich, Munich, Germany; 81 Swiss Institute of Allergy and Asthma Research (SIAF), University of Zurich, Christine Kühne-Center for Allergy Research and Education (CK-CARE), Davos, Switzerland; 82 Allergy and Immunology Unit, Fondazione ‘Salvatore Maugeri’, Pavia, Italy; 83 University of Ferrara at St. Anna Hospital, Ferrara, Italy; 84 Internal Medicine Pad Maragliano, Genoa, Italy; 85 Center for Rhinology and Allergology Wiesbaden, University Hospital Mannheim, Mannheim, Germany; 86 Dietetics and Nutritional Science Dept, Harokopio University, Athens, Greece; 87 Hospital La Paz Institute for Health Research, Madrid, Spain; 88 Institute for Prevention and Occupational Medicine of the German Social Accident Insurance, Allergology/Immunology; Ruhr-University, Bochum, Germany; 89 School of Life Sciences, Northumbria University, Newcastle upon Tyne, United Kingdom; 90 Hospital Divino Espirito Santo de Ponta Delgada, Ponta Delgada, Portugal; 91 ENT Department, University Hospital Split, Split, Croatia; 92 Hospital Civil, Malaga, Spain; 93 Nr. 101 - Odilija Rudzeviciene, Vilnius University, Vilnius, Lithuania; 94 Ludwig-Maximilians-Universität, Munich, Germany; 95 Center of Allergy & Immunology, Tbilisi, Georgia; 96 Complesso Integrato Columbus, Rome, Italy; 97 Department of Pediatric Allergy, Hacettepe University, Ankara, Turkey; 98 Department of Pediatric Allergy, King's College London, MRC & Asthma UK Centre for the Allergic Mechanisms of Asthma, London, United Kingdom; 99 Immunoallergology Department, Coimbra University Hospital, Coimbra, Portugal; 100 Rimini Infermi Hospital, Rimini, Italy; 101 Greifswald University Medical School, Greifswald, Germany; 102 Klinikum rechts der Isar der TU München, ZAUM, Munich, Germany; 103 Clinic for Rheumatology and Clinical Immunology/Allergology, University Hospital of Bern, Switzerland, Bern, Switzerland; 104 University of Edinburgh, Edinburgh, United Kingdom; 105 Azienda Ospedaliero-Universitaria, Verona, Italy; 106 Institute of Technology, Tartu University, Tartu, Estonia; 107 Catholic University of Leuven, Leuven, Belgium; 108 Occupational Medicine, Terni Hospital, University of Perugia, Perugia, Italy; 109 National Institutes of Health, Bethesda, MD, 20892, USA; 110 Centre Hospitalier Universitaire Vaudois, Lausanne, Switzerland; 111 Department of Experimental Dermatology and Cosmetology, Jagiellonian University, Krakow, Poland; 112 Emma Children's Hospital Academic Medical Center, Amsterdam, The Netherlands; 113 Department of Dermatology, Medical University of Graz, Graz, Austria; 114 Department of ENT and Pediatrics, Academic Medical Center, Amsterdam, the Netherlands; 115 Dept. of Otolaryngology-Head and Neck Surgery, Temple University, Philadelphia, USA; 116 The David Hide Asthma and Allergy Research Centre, Isle of Wight, England, United Kingdom; 117 St James’s Hospital, England, United Kingdom; 118 Charité - Universitaetsmedizin Dpt. of Dermatology, Berlin, Germany

**Keywords:** Allergy, Allergic diseases, Policy, Research needs, Research funding, Europe

## Abstract

In less than half a century, allergy, originally perceived as a rare disease, has become a major public health threat, today affecting the lives of more than 60 million people in Europe, and probably close to one billion worldwide, thereby heavily impacting the budgets of public health systems. More disturbingly, its prevalence and impact are on the rise, a development that has been associated with environmental and lifestyle changes accompanying the continuous process of urbanization and globalization. Therefore, there is an urgent need to prioritize and concert research efforts in the field of allergy, in order to achieve sustainable results on prevention, diagnosis and treatment of this most prevalent chronic disease of the 21^st^ century.

The European Academy of Allergy and Clinical Immunology (EAACI) is the leading professional organization in the field of allergy, promoting excellence in clinical care, education, training and basic and translational research, all with the ultimate goal of improving the health of allergic patients. The European Federation of Allergy and Airways Diseases Patients’ Associations (EFA) is a non-profit network of allergy, asthma and Chronic Obstructive Pulmonary Disorder (COPD) patients’ organizations. In support of their missions, the present EAACI Position Paper, in collaboration with EFA, highlights the most important research needs in the field of allergy to serve as key recommendations for future research funding at the national and European levels.

Although allergies may involve almost every organ of the body and an array of diverse external factors act as triggers, there are several common themes that need to be prioritized in research efforts. As in many other chronic diseases, effective prevention, curative treatment and accurate, rapid diagnosis represent major unmet needs. Detailed phenotyping/endotyping stands out as widely required in order to arrange or re-categorize clinical syndromes into more coherent, uniform and treatment-responsive groups. Research efforts to unveil the basic pathophysiologic pathways and mechanisms, thus leading to the comprehension and resolution of the pathophysiologic complexity of allergies will allow for the design of novel patient-oriented diagnostic and treatment protocols. Several allergic diseases require well-controlled epidemiological description and surveillance, using disease registries, pharmacoeconomic evaluation, as well as large biobanks. Additionally, there is a need for extensive studies to bring promising new biotechnological innovations, such as biological agents, vaccines of modified allergen molecules and engineered components for allergy diagnosis, closer to clinical practice. Finally, particular attention should be paid to the difficult-to-manage, precarious and costly severe disease forms and/or exacerbations. Nonetheless, currently arising treatments, mainly in the fields of immunotherapy and biologicals, hold great promise for targeted and causal management of allergic conditions. Active involvement of all stakeholders, including Patient Organizations and policy makers are necessary to achieve the aims emphasized herein.

## Background

Allergies represent the most frequent chronic diseases in Europe today, affecting, with the most conservative estimates, the daily lives of more than 60 million people. While at the beginning of the 20^th^ century, allergies were viewed as rare diseases, the last few decades have seen a dramatic increase in disease burden. The industrial and technological revolution has led to environmental changes, including climate variation, pollution and microbial sterilization, but also to an urban, sedentary life style, affecting on one hand the intensity, type and diversity of external exposures, while on the other hand altering the normal immune/inflammatory responses.

Allergies involve almost every organ of the body in variable combinations with a broad spectrum of possible symptoms, and thus their manifestations cover a wide range of phenotypes. Studies in Europe have shown that up to 30% of the population suffers from allergic rhinoconjunctivitis, while up to 20% suffer from asthma and 15% from allergic skin conditions [1,2]. These numbers match those reported for other parts of the world, such as the USA and Australia. Food allergies, are becoming more frequent and severe; occupational allergies, drug allergies and allergies to insect stings (occasionally fatal), further aggravate the burden of the allergy epidemic. In contrast to the popular belief that allergies are mild conditions, a considerable and increasing proportion of patients (15%-20%) have severe, debilitating disease and are under constant fear of death from a possible asthma attack or anaphylactic shock [3]. Within the EU, there are nevertheless wide geographical variations in the incidence of allergies with a south to north and east to west gradient [4,5]. An alarming observation is that most allergic conditions start in childhood and peak during highly productive years of individuals, with allergic rhinitis affecting up to 45% of 20–40 year old Europeans. The numbers may even be an underestimation, as many patients do not report their symptoms or are not properly diagnosed. Indeed, it is estimated that approximately 45% of patients have never received a diagnosis [6]. Notwithstanding evidence suggesting a plateau in some areas, the European Academy of Allergy and Clinical Immunology (EAACI) warns that in less than 15 years more than half of the European population will suffer from some type of allergy!

### Major knowledge gaps in allergy

• Causes of allergy, as well as reasons for recent increase in prevalence are unknown

• The natural history, including mechanisms of spontaneous resolution, are unknown

• There is marginal understanding of interactions among microbes, immune system and allergic disorders

• Therapeutic targets with potential for a complete cure are scarce

Independent of incidence, age group or nationality, it is important to realize that allergic diseases have a detrimental impact on the quality of life of patients and their families, affecting their personal development, career plans and lifestyle choices. Allergies may affect sleep and mood, school or work competence, and social interaction [7]. For example 43% of patients with allergic rhinoconjunctivitis have sleep disturbances and 39% have difficulty in falling asleep [6]. The possibility of failing an examination increases by 40%-70% in school-age children with rhinitis [6]. Allergic individuals have a higher risk of developing depression [7]. The impact of allergies on quality of life can be as high or even higher than that of diseases commonly perceived as being more ‘serious’ [6,8].

At the society level, the rising prevalence of allergic diseases poses a multifaceted, major socioeconomic burden on national and European budgets. The increased use of health services, hospitalization and pharmaceutical costs, in addition to the billions of days of lost productivity through absenteeism or presenteeism (people going to work but being unable to perform), reveal a worrying prospect for public health when it comes to allergies [9]. It is estimated that the annual cost of asthma in Europe is over €18 billion [8]; allergic rhinitis may cost several times more (up to €100 billion, according to unpublished data from the Global Allergy and Asthma Network of Excellence, GA^2^LEN, investigators). Skin allergy care costs may be as high as that of asthma [10,11]. Given the nature of current lifestyles, the ageing population and continuing environmental changes, these numbers are likely to increase, unless a concerted effort is devised to understand the causes and mechanisms of allergy and design effective strategies for prevention and/or treatment.

Special attention should be paid to childhood allergies as these usually demonstrate a persistent and varying course: many children first develop eczema, followed by asthma and allergic rhinitis, the so called “allergic march”. Therefore, early diagnosis and adequate control of allergy is crucial especially for children. The Polish Presidency of the Council of the EU underlined this problem in its conclusions on “Prevention, early diagnosis and treatment of chronic respiratory diseases in children” (unanimously adopted by the EU Ministers of Health in December 2011) [12].

EAACI is the leading professional organization in the field; its members are nearly 8000 physicians, researchers and academicians, as well as all of the European National Allergy Societies. EAACI is dedicated to improving the health of people affected by allergic diseases by promoting adequate patient care, advancing basic and clinical research and encouraging education and training. The European Federation of Allergy and Airways Diseases Patients’ Associations (EFA) is a non-profit network of allergy, asthma and chronic obstructive pulmonary disorder (COPD) patients’ organizations, representing 35 national associations in 22 countries and over 400,000 patients. EFA is dedicated to making Europe a place where people with allergies, asthma and COPD have the right to the best quality of care, a safe environment, the right to live uncompromised lives and be actively involved in all decisions influencing their health. More information about the organizations and their programs can be found at http://www.eaaci.org and http://www.efanet.org.

Following their missions, EAACI and EFA, advocate for feasible, sustainable and patient-centered strategies for allergy research. There is currently a great need for a sustained investment in allergy research. Key mechanisms need to be further understood. Several important findings at the basic immunological level are close to being translated into bedside treatments. New preventative approaches require large-scale studies to be confirmed so that they can be used for public health purposes.

This article, written by Section and Interest Group Board members of EAACI and co-authored by EFA, intends to create a milestone, describing the current research needs in the wide spectrum of allergic diseases, and provide key recommendations to provide input for consultations on current and future research programs at the national and European levels. By prioritizing various projects of basic, translational and clinical allergy research, and efficient networking, such programs may not only yield ground-breaking results, but also crucially inform patient and public health strategies, reducing health-related costs and improving the quality of life of millions in Europe and around the world, while providing opportunities for a strong European Research Area and an innovative knowledge-based European industry. They can also inform novel National Plans following the model of the Finnish Asthma and Allergy Programs [13] , implementing wide stakeholder participation.

## Objectives and methodology

The aim of this article is to briefly describe the current understanding of the whole spectrum of allergic diseases and conditions, and to identify the immediate knowledge gaps amenable to research at the national and European levels. Input was obtained by all EAACI Sections and Interest Groups; the Chair, Secretary and Board Members of each group prepared a paragraph related to their specific domain. EFA was also consulted and provided input to the text. These were subsequently integrated and reviewed by the EAACI Executive Committee members, who provided by consensus a concluding part and summary of key points.

## Understanding the allergy epidemic

### Epidemiology

#### Research needs in the epidemiology of allergy

• Europe-wide epidemiological studies on occupational allergy, drug allergy, venom hypersensitivity, exposure to various environmental agents

• Interactions between various risk factors of allergy development to explain prevalence differences

• Link between sensitization and clinical allergy development

• Incidence, prevalence studies of various disease phenotypes and endotypes

• Development of pan-European registries

Epidemiology is the study of health and disease in populations. Several landmark studies have defined the global burden of allergic diseases, and have demonstrated that the problem is growing. Major demographic, socio-economic and environmental developments such as urbanization, globalization, upcoming economies (BRIC countries) and climate change will most likely contribute to further increases. The International Study of Asthma and Allergies in Childhood (ISAAC) reports that well over 20% of children in European countries suffer from an allergic disease at some point during their childhood [14]. This study also assessed allergy prevalence sequentially and observed a rise in prevalence especially in areas where allergic diseases were previously less common [2]. Large-scale population-based data from the European Community Respiratory Health Survey (ECRHS) [15] and the Global Allergy and Asthma Network of Excellence (GA2LEN) [16] clearly show that allergic diseases are significant health problems in adults too. A similar initiative for food allergy (EuroPrevall) [17], has been recently completed, but population-based information is currently lacking for occupational allergy, drug and insect venom hypersensitivity, as well as daily exposures to environmental agents such as pollutants and cosmetics. These studies have revealed significant differences in allergy prevalence even amongst those of the same ethnic background, advocating that, notwithstanding the importance of genetic factors, environmental factors are likely to be responsible for the observed prevalence gradients and time trends. Socio-economic background, family size, urban dwelling, farm exposure, infection history, diet, obesity, use of certain drugs, tobacco smoke exposure and indoor and outdoor air pollution are among the factors associated with atopic disease.

In spite of these clear indications, we are currently missing studies that aim at explaining the prevalence differences and trends between populations taking *all* known risk factors into account as well as the interactions between risk factors (including gene-environment interactions). Rapid developments in the field of information technologies allowing systems approaches now offer the opportunity to study these complex issues in a comprehensive way.

When it comes to allergies, it is vital to maintain an open mind about the nature of causal influences and be prepared to follow up clues or signals emerging even in, at first sight, unlikely places: for instance, the protective effect of Alpine farms has been an important stimulus to aetiological enquiry [18]. In addition, the link between allergic sensitization and clinical allergy remains poorly understood and new molecular methods may shed further light on this fundamental issue.

One limitation of large population-based studies has been the use of questionnaires in the diagnosis of asthma or allergic rhinitis and that more objective markers, such as spirometry and bronchial hyper responsiveness, do not capture the episodic nature of the disease and often do not correspond to the clinical phenotype. An advantage in atopic dermatitis is that the skin is readily accessible to examination. The strong association between clinical atopic dermatitis and filaggrin skin barrier gene mutation carriage has opened up new avenues to explore gene-environment interactions, not only in the context of atopic dermatitis, but also allergic respiratory disease and food allergy [19].

Another important area of epidemiological research is the application of epidemiological methods to explore the quality and safety of medical care provided for patients with allergic diseases, for instance through establishing registries with a main focus on the efficacy and safety of treatments, such as immunotherapy, systemic immunosuppressive drugs and biologicals. Registries should also capture trends in disease prevalence and severity and differences between age and/or social groups. Data on emerging allergens, including work-related exposure, could also be monitored.

Overall, we need to find better ways of identifying the phenotypes of allergic disease, using standardized methodology between studies to facilitate direct comparability. This will then allow us to revisit known and hitherto unexplored risk and protective factors, preferably in longitudinal population-based cohorts that take into account environmental, genetic and immunological factors (biomarkers) as well as the fluctuant nature of allergic diseases and disease severity. Novel approaches to genetic and gene-environmental interaction studies will be helpful to identify the ‘missing heritability’.

### Immunology, molecular and cellular mechanisms

#### Major gaps in understanding the allergic immune response

• Immunological basis of allergy epidemic

• Innate immune response to molecules that are coexposed with allergens

• Role of novel subsets of T cells, B cells and innate lymphoid cells in allergy development

• Epithelial barrier function and its role in allergy development and chronicity

• Mechanisms of development of immune tolerance to allergens and novel ways to induce this

• Understanding epigenetic regulation of the allergic inflammation

• Development of novel biologicals to treat allergy

• Identification of novel biomarkers for endotyping patients for the prediction of treatment response and prognosis

• Development of immunological registries and Europe-wide disease-specific biobanks

In diseases involving the immune system such as allergy, autoimmunity, transplantation rejection, cancer and infections, antigens are either the direct or indirect cause of the disease and can be targeted for treatment [20]. The investigation of what makes a protein an allergen has been a prerequisite of understanding allergic disease to develop strategies for immune intervention. Allergens are almost always proteins, but not all proteins are allergens. A protein with allergenic activity should display two properties a) induction of IgE response, which involves the sensitization phase including T cell, B cell and dendritic cell cooperation, and b) induction of a clinical response to the same or similar protein on subsequent exposures, which involves immediate and late phase responses [21]. Many allergens contain potent stimulatory properties for the epithelium, such as the protease activity of house dust mite. Besides proteases and oxidases, extracts of pollen contain low molecular weight molecules such as pollen-associated lipid mediators or adenosine exhibiting a potential to stimulate and modulate immune cells [22]. Therefore, when exposed to e.g. pollen, it is more than just allergenic proteins that we inhale. More knowledge on the immune stimulatory properties (adjuvant-like activity) of allergens, in addition to their antigenic potential will be of great importance and might help in modifying allergens in the application of allergen specific immunotherapy.

**Figure 1:**
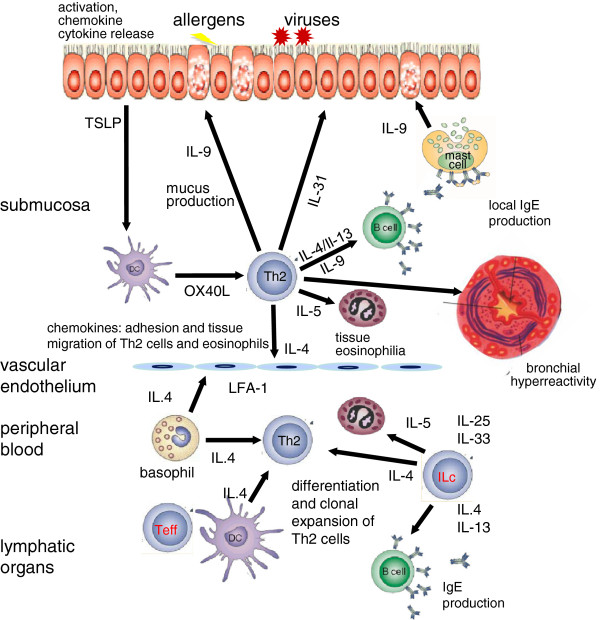
**Mechanisms of allergic inflammation in asthma.** Epithelial cell activation by allergens, viruses, bacteria and pollutants takes place and their proinflammatory cytokines and chemokines induce inflammation and contribute to Th2 response with TNF-α, IL-13, TSLP, IL-31, IL-33. Th2 response involves multiple cytokines such as IL-4, IL-5, IL-9, IL-13, IL-25, IL-33, eosinophilia, and local and systemic IgE production. A series of chemokines are produced and migration of inflammatory cells to the allergic tissues takes place. In addition, other effector T cell subsets, such as Th9, Th17 and Th22 cells play inflammatory roles. Cross-linking of IgE receptor FcεRI on the surface of mast cells and basophils and their degranulation, induces a type 1 allergic response. The activation of smooth muscle cells, myofibroblasts, angiogenesis and subepithelial fibrosis, lead to remodeling. Bronchial hyperreactivity takes place, with enhanced susceptibility to bronchoconstriction. Innate lymphoid cells may contribute to many aspects of allergic inflammation by the help of multiple cytokines. Epithelial apoptosis and shedding are essential in the mechanisms of eczema and asthma [23,24]. Survival and reactivation of migrating inflammatory cells and their interaction with resident tissue cells and other inflammatory cells augment allergic inflammation.

An increasing body of evidence indicates that immune responses in allergy involve a comprehensive network of cellular and molecular interactions (Figure 1). Novel insights from implicated environmental influences on the development of allergy have increased interest in innate immune responses preceding and directing the adaptive immune response. In parallel, we have now identified a role of the local tissue immune response, not only in determining the implication of a specific tissue in allergy (i.e. atopic asthma vs. atopic dermatitis) but also the role of structural cells such as epithelial cells in the innate immune responses to environmental triggers, including allergens.

As a direct result of epidemiological findings of reduced incidence of allergy in farm environments, and many others on the so-called hygiene hypothesis, there has been interest in the development of early and innate immune responses. Pattern recognition receptors such as Toll like receptors (TLR) are important in these reactions, essentially deciding over the pro- or anti-inflammatory nature of subsequent adaptive immune responses. It is becoming clear that these receptors do not only play a role on innate immune cells directing their response to e.g. lipopolysaccharide, double stranded RNA or bacterial DNA, but also on tissue cells such as epithelial cells or keratinocytes [25]. Very recently, a novel category of innate lymphoid cells with still a wide variety of names, such as nuocytes, lymphoid tissue–inducer cells (LTi cells) or innate lymphoid cells (ILCs) have been described. The demonstration of IL-13 and IL-5 production by so-called type-2 ILCs in allergic inflamed tissue is a strong hint that these cells can have an important role in the Th2-type skewing in allergic tissues, even in the absence of direct involvement of antigens [26]. The epithelial cells at environmental interfaces (skin, respiratory tract and gut mucosae) are also important for allergy development, possibly through reduced barrier function and/or the inherent properties of epithelial cells to induce Th2 responses [27]. There is increasing evidence that the composition of the microbiota of the gut (and possibly of the airways or even skin) play an important role in deciding over development towards allergy or protection against it [28]. The epigenetic regulation of allergic inflammation rendering certain genes or gene areas more accessible to translation should be extensively studied and is expected to provide better links to the influence of the environmental changes. Also, multigenerational epigenetic memory can help us to explain the inherited influence of the environmental exposure [29].

In addition to the almost classical definition of T helper cells in Th1 and Th2, many more T cell subsets have recently been described, including regulatory T cells, Th17, Th9 and Th22 cells [30-33]. Moreover, for all T cell subsets it is evident that their role can differ in different tissue types. Accordingly, there is increasing evidence that differentiated Th cell populations can alter the range of cytokines they produce, indicating a certain degree of “cell plasticity”. Understanding the molecular basis of the process through which cells modify their cytokine-producing potential is likely to provide interesting insights that may allow the development of strategies to alter Th cell function in immune-mediated pathologies, including allergic diseases. Amongst T-cell subsets, it is becoming clearer that CD8 T cells, classically called cytotoxic T cells, contain potent immunomodulatory subsets, which may be potent in the release of several cytokines, including IL-13. In addition, the immune regulatory and immune suppressive roles of various T and B cell subsets, such as T regulatory cells and B regulatory cells still remain to be elucidated.

In many inflammatory diseases, application of biologicals is an important option in treating severe forms of the disease. It is surprising that there is only limited potential of the known biologicals developed specifically for allergy [34]. Anti-IgE has major IgE decreasing effects, but is only indicated in a small subset of allergic asthmatics [35] at present although licensing studies for chronic urticaria are in an advanced stage. Blocking eosinophil differentiation and activation by anti-IL-5 is effective *in vitro,* but results from clinical studies [36], suggest the need for identifying responsive populations. The outcome of the application of these agents has indicated that our knowledge of the immunological mechanisms responsible for allergic inflammatory diseases remains incomplete. Although improved forms of anti-IgE or blocking of IL-5 effects may provide better drug efficacy, it is also important to design new biologicals and find novel application of established ones. Combined application of allergen-specific immunotherapy (SIT) with anti-IgE treatment, might improve safety and efficacy of immunotherapy.

SIT is the only treatment that alters the immunological basis of the disease. The immune responses to allergen observed during SIT are being described at increasing level of detail, but there is a continuous debate around mechanisms of induction and maintenance of tolerance. It is absolutely essential to further elucidate the immune mechanism of SIT. Only in this way new leads can be found to transform SIT from a therapy requiring “chronic administration” for years in a row into an effective therapeutic vaccination based on a small number of shots, and perhaps even a preventive vaccination [34].

Evidence is gathering that the complex interplay between the innate and adaptive immune response both of the myeloid and lymphoid lineages, in combination with the immune response of the tissue ultimately determines the development and expression of allergic diseases. A further important issue comprises adjuvant factors from the allergen carriers. We face an interesting and challenging era that will implicate all novel immunological insights in understanding better the immune-pathophysiological mechanisms in allergic diseases. This will lead to improved insight in the disease itself and also in new treatment options that may even result in allergy prevention, which is a major concern not only for parents thinking about their children, but for society as a whole.

### Diagnostics

#### Next steps in allergy diagnosis

• Improvement of molecular diagnostics methods

• Diagnosis of biomarkers for endotyping

• Easy and standardized tests for cellular diagnosis

• Development of point of care assays and devices

• Development of tests for endotyping, follow up of exacerbations and treatment response

The challenges in allergy diagnosis lie in developing methods that are objective, rapid, reproducible, cost-effective, sensitive and specific for the disease. They need to be able to identify exposures and contribute to decision making for treatment, preferably also in primary care. In addition, tests monitoring the response to treatment and allowing the comparison of preclinical and clinical effects across large cohorts and in several countries are needed. As recombinant molecular therapies are becoming available, there is a need to tailor treatment to disease burden, phenotype and/or endotype more effectively, which has always been a key request by patient groups; in this respect improved allergy diagnosis is a prerequisite. Molecular diagnostic methods enable a much more detailed analysis of the allergen molecules and their association with clinical presentations. Molecular approaches have great potential to significantly improve the prognostic value of diagnostic tests, but large-scale clinical studies are needed for their validation. Both allergen molecules and recombinant antibodies are made available to enlarge the repertoire of diagnostic tests [37]. The first allergen molecule-based multiplex tests have reached the market [38], significantly increasing diagnostic and epidemiological options [39].

Improvement and standardization of cellular tests, such as the basophil activation test, [40] will enable laboratories to more closely monitor disease status and advance allergy diagnosis and monitoring from the mere assay of IgE affinity, concentration and avidity to an integrated evaluation of the burden of allergic disease on the individual. Such an approach is not feasible at the moment. A similar methodology to the one used to produce standardized T cell tests, should be adopted. The involvement of reference centers and standardized comparisons of efficacy can lead to standardized methods of analysis. In combination with molecular tools, this will enable improved-resolution diagnosis with a higher prognostic value, which in turn will result into more precise diagnosis and superior monitoring of allergic disease. Recent advances in multicolor flow-cytometry are expected to contribute to cellular diagnosis and disease phenotyping/endotyping. In addition, detection of recently discovered microRNAs and/or exosomes in body fluids may open new ways for non-invasive diagnosis and monitoring of patients. Development of a standardized mast cell line similar to resident tissue mast cells would revolutionize the understanding and diagnosis of mast cell-mediated immediate hypersensitivity reactions.

In the diagnosis of drug allergy, cellular tests can be more useful both for diagnosis of type I allergy [41] and for diagnosis of the more severe type IV allergy [42]. As treatment options expand and life expectancy increases, people will require more frequent medical treatment and ex vivo diagnostic approaches will become more important.

Ex vivo tests for type I allergy will play a role in both the diagnosis of occupational allergy and food allergy. Moreover, they are an important element in diagnosis of paediatric allergy, since they constitute an alternative to allergen exposure tests currently considered as the gold standard in diagnosis of allergy.

In the near future, individualized –omics analyses will provide a wealth of detailed information.

A new integrated approach, combining data of genomics (genome-wide SNP association studies) and metabolomics with disease-specific proteome and transcriptome data from biopsies and body fluids (blood, saliva, nasal lavage) will allow a comprehensive characterization of complex diseases for the first time. Furthermore, diagnostic tools to characterize the environment such as microbiome, pollen counts, mite counts, allergen quantification, mould counts, smoke, and other pollutants need to be developed and standardized across Europe.

In addition to diagnostic approaches based on large laboratory-intensive methods, individualized point of care testing should be developed as this can/will remove a burden from the health sector with patients eventually capable of partly monitoring their own allergic disease and optimizing their treatment in order to rely more on a predefined plan and electronic support (ICT and e-health) and less on frequent visits to the allergy clinic.

### Patient-reported outcomes

Patient-reported outcomes (PROs) refers to all health-related reports coming from the patient, without involvement or interpretation by physician or others [43] (i.e. Health Related Quality of Life (HRQoL), symptoms, illness perception, satisfaction, well-being, perceived disease control).

PROs recently gained great attention in clinical research and by regulatory bodies due to their importance in the overall treatment efficacy assessment [44-46].

The role of patient’s perspectives is now underlined by the GRADE system [47], which also includes patients’ preferences and values as cornerstones in the process of formulating recommendations towards diagnostic and therapeutic interventions, thereby contributing to the translation of scientific research into real life.

A critical aspect in the management of allergic diseases is their impact on subjective experience. Available data show that, from the patients’ perspective, allergy is more than just an annoying disease: when compared to healthy subjects, patients with asthma, rhinitis, chronic urticaria, atopic dermatitis and food allergy reported markedly reduced HRQoL [48]. The burden of the disease, besides functional and practical problems, includes some emotional aspects: the presence of a chronic condition, the need to take medication and change habits and lifestyle may cause anxiety, tension and irritability and an unsatisfactory social life.

Among PROs, HRQoL and patient-reported symptoms have been extensively evaluated in asthma and rhinitis [49,50], and more recently, also in chronic urticaria and food allergy [51].

In contrast to the advanced stage of research in respiratory and skin allergy, little is known about HRQoL in allergic conditions such as drug hypersensitivity, occupational allergy and insect venom allergy.

Although some evidence about subjective view of allergic diseases and their treatment has been achieved, further unmet needs and unexplored areas should be underlined. First, there is a need to develop clinical trials in which PROs are the primary or co-primary outcome. Another point is the necessity to assess patients’ viewpoint with a rigorous methodological procedure (use of validated tools, correct administration of the questionnaires and report of complete results).

Furthermore, there is a need to reach a global picture of patient’s perspective about allergy and its treatment by exploring the following uncovered areas: other PROs (satisfaction, preferences, well-being, illness perception) besides HRQoL; burden of comorbidity on PROs; specific population (children, adolescents, elderly, parents of allergic children); relation of PROs with other clinical measures of health impact; relationships among different PROs and between PROs and psychological variables.

Overall, there is a need for a more correct and extensive assessment of PROs, both in clinical trials and in routine practice, to capture information unavailable from other sources, which is crucial for predicting health outcomes, for establishing health policy and for the optimal management of allergic diseases.

Age-specific tools for patient information, education and peer support, major objectives of patient organizations, should be further developed.

### Chronic Respiratory Allergies

In the last decade, it has been clearly demonstrated that allergies of the respiratory tract (asthma and rhinitis) very frequently coexist in the same patient, have similar epidemiology, share mechanisms and interact in terms of treatment and risk for persistence. Therefore, asthma and rhinitis are currently considered as part of a common syndrome, for which different terms, including ‘chronic respiratory allergy’ have been proposed. Additionally, the most common forms of allergic conjunctivitis occur together with rhinitis, in which case, allergic rhinoconjunctivitis is the prevailing term.

EFA’s 4-year Respiratory Allergies Project ‘Raise Awareness, Relieve the Burden’ aims at increasing the awareness of allergy as a serious chronic disease, to receive earlier concrete diagnosis with proper management, to avoid the increase of severity and to relieve the burden that it may impose on the lives of affected people. EFA published the Book on Respiratory Allergies – ‘Raise Awareness, Relieve the Burden’ in 15 languages and launched a “Call to Action” at the European Parliament (EP) to call upon policy makers to act in this crucial topic [6].

### Asthma

Asthma is a major global health problem contributing greatly to socio-economic burden: more than 200 million people of all ages suffer from asthma worldwide [52], and 250,000 people die prematurely every year due to asthma. Although the majority of patients with asthma can potentially be sufficiently controlled using currently available treatments, present management practices have considerable limitations in a relatively small, but important subgroup of patients with severe asthma, who suffer from ongoing symptoms, frequent exacerbations and reduced quality of life, despite receiving the best available treatment. So, although this patient group comprises about ~10% of the overall asthmatic population, it accounts for more than 75% of the costs attributable to asthma. Already in 2003, the total cost of asthma in Europe was calculated at 17.7 billion EUR per year, and productivity lost to patients’ poor control of their asthma was estimated at 9.8 billion EUR per annum [8]. The necessity to improve the management of asthma has frequently been underlined [53].

#### Major unmet needs in chronic respiratory allergies

• Mechanisms and management of severe cases

• Characterization of phenotypes and identification of novel biomarkers for endotypes

• Devise a consensus definition for severe asthma and exacerbations

• Identification of factors increasing the risk of asthma exacerbations and preventing them

• Vaccine development against viruses that trigger exacerbations and severity

• Patient-tailored treatment

• Development of biologicals

• Establish European registry and biobank for respiratory allergies in meticulously phenotyped patients

**Figure 2:**
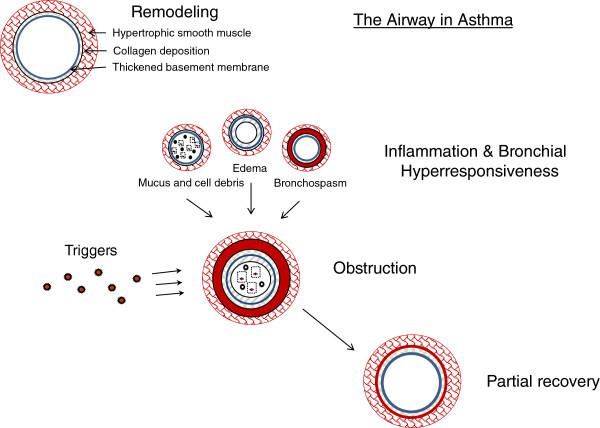
**Pathophysiology of asthma at steady state and during an exacerbation.** The airways of asthmatic individuals are characterized by pathological changes, including thickened basement membrane, collagen deposition and hypertrophic smooth muscle, collectively called ‘airway remodeling’. Inflammation is triggered by a variety of factors, including allergens and respiratory viruses. These factors also induce hyperreactive responses in the asthmatic airways, associated with mucus and cell debris released into the lumen, oedema and bronchoconstriction, leading to airway obstruction and related acute exacerbations. Although pathophysiological changes related to asthma are generally reversible, recovery may be partial.

An additional significant unresolved problem in the management of asthma is the failure to prevent and/or promptly treat asthma exacerbations, which are associated with significant morbidity, risk of death and high treatment cost [54] and whose prevention in children may reduce the risk of subsequent adulthood asthma [55] (Figure 2). Identifying the factors increasing the risk of asthma exacerbations and designing novel treatments that reduce the risk are key priorities. Virus infections have a major role in asthma exacerbations and preventive vaccines or passive immunizations are awaited to prevent virus infection-induced exacerbations. In addition, to allow for a rational approach to the management of asthma, we must consider the emerging evidence for pathophysiological heterogeneity of the disease, particularly in its more severe forms. The conceptual framework of asthma endo/phenotypes developed by the EAACI [56,57] can offer the basis for an improved perception of the pathophysiological mechanisms involved in severe asthma and asthma exacerbations and enable target identification and development of novel treatments. Prediction of the outcomes and personalization of treatment through innovative approaches and new technologies can contribute to an effective intervention in terms of prevention and reduction of healthcare costs, with improved quality of life for asthma patients [58]. So, asthma research should aim to implement a consensus definition of severe asthma and asthma exacerbations that could usefully guide treatment and match this with diagnostic tools for “scoring” the asthma patient in terms of disease severity and future risk for the use in primary care. The recognition of asthma as a heterogeneous disease is a prerequisite for planning studies aimed at differentiating between different endo/phenotypes and the sequential creation of robust clinically-relevant and endo/phenotype-specific biomarkers and therapies targeting specific pathophysiology, including longitudinal outcomes. In this effort, we must also identify relevant environmental factors and the influence of current and prior medication. The accomplishment of the above aims also necessitates the establishment of large Research Tissue Banks (including DNA, serum, lavage fluids and other biological samples) in meticulously clinically phenotyped patients and the execution of clinical studies to improve the evidence-base, since most randomized, controlled trials to date have been carried out in highly selected patients; we should now aim for effectiveness, rather than efficacy studies. 

### Rhinitis

Allergic rhinitis affects up to one third of the adult population, hence having a major socio-economic impact. In spite of the high prevalence of the disease and the availability of effective and safe treatments [59], a significant number of patients remain deprived of an accurate diagnosis and specific anti-allergic treatment [60], while another group of patients remain symptomatic in spite of adequate medical therapy [61].

There is a need for the better characterization of the different phenotypes of allergic rhinitis, which should be matched with improved understanding of both the environmental and occupational factors determining severity of disease and the endogenous and exogenous factors that contribute to the progression from rhinitis to asthma in childhood and adolescence. In this respect, we must also define control of the disease, through improving insights into the mechanisms underlying uncontrolled allergic rhinitis. The similarities and differences between rhinitis and asthma should be further characterized [62]. Diagnosis-wise, there is a need for objective evaluation tools of disease severity and impact [63]. Treatment-wise, we need to both define patient-related reasons for undertreatment, reasons for underdiagnosis and non-compliance of patients to treatment and therefore design patient-oriented treatment strategies for optimal efficacy of treatment and compliance to therapy [64]. One aspect, which also requires attention, is the role of complimentary/alternative medicine in allergic rhinitis, an approach that is widely used and is a common question from patients.

### Ocular allergies

Ocular allergies have been studied to a lesser extent, compared to other allergies. The evaluation of prevalence and severity range of ocular allergies in Europe requires additional effort. The more serious forms, such as allergic keratoconjunctivitis (AKC), one of the most serious complications of atopic dermatitis, and vernal keratoconjunctivitis (VKC), which may have serious ocular complications such as keratoconus, cataract and retinal detachment, are of particular interest. Pharmacological and immunological research is needed to evaluate new possibilities in modifying the ocular immune response in these diseases. Although evidence on ocular immunopathology is available, no comprehensive solution exists.

The roles of allergens and cross-reactivity, as well as criteria for consequent immunotherapy, are well established in seasonal allergic rhinoconjunctivitis, but far from clear in perennial conjunctivitis, AKC and VKC. Phenotyping and genotyping of these diseases is needed. This will allow us to effectively choose treatments (pharmacological and/or allergen immunotherapy).

Furthermore, evidence-based recommendations for use of available treatment options in ocular allergy are needed.

### Dermatological allergies

The skin plays a major role in allergy by acting as the first frontier of the body to allergen contact and sensitization. An intact epidermal barrier protects the immune system from exposure to exogenous allergens, whilst an impairment of the skin barrier, either mechanically or functionally, allows allergen penetration into the sub-epidermal layer and promotes sensitization. The skin as route of sensitization is no longer seen as necessarily being limited to allergy of the skin, but is more and more considered as a route of sensitization for allergy involving other organs.

The atopic march starts in newborn skin. The role of skin integrity and skin microbiota in childhood as interventional targets to prevent atopic sensitizations should be studied in large-scale studies with well-defined populations [65]. The role of allergen avoidance versus early allergen ingestion in promoting tolerance versus sensitization to food allergens and subsequent atopic symptoms must be evaluated [66]. The possibilities of tolerance induction to prevent symptoms caused by food allergens for both children and adults should be studied in more depth, with new monitoring methodologies. There is an opportunity to study the effect of newer biological drugs on prevention of the atopic march.

#### Major unmet needs in skin allergy

• Identification of molecular, genetic and environmental determinants of the atopic march

• Targeting skin barrier function for novel treatments and prevention

• Role of skin microbiota and colonizing pathogens

• Better characterization and identification of novel biomarkers for endotypes

• Monitor new contact sensitizers and promote patch testing

• Develop and evaluate new drug classes including biologicals

• Epidemiological monitoring of urticaria and hereditary angioedema

Atopic dermatitis is one of the most common chronic inflammatory skin diseases with an estimated prevalence of 20% in children and 5% in adults [67,68]. The direct and indirect costs of atopic dermatitis are significant and comparable to other common chronic diseases with large annual economic burdens. However, compared to psoriasis, the most closely analogous complex inflammatory skin disease, treatment options are limited. There is a need for new classes of drugs, such as biologicals for treating it, including those targeting the immune system, skin barrier, and skin microbiota. Developing methods for improving the skin barrier function should be an important focus of future research [69]. It is increasingly clear from advances in molecular genetics that a close interaction and reciprocal regulation of epithelial function by immunological events underlie allergic disease, and epithelial barrier function is becoming more and more important in other forms of allergic diseases, such as asthma and rhinitis [70]. A bench to bedside approach is necessary to identify the key elements of these interactions systematically.

Allergic contact dermatitis is one of the most common work-related diseases and is of appreciable public health importance because of its significant economic impact. As knowledge on its diagnostics through patch testing is waning, promoting patch test education is a paramount issue. New contact sensitizers continue to appear [71] and older ones become important in new situations, emphasizing the need to monitor their appearance in Europe and worldwide and integrate new knowledge to relevant regulations at all levels. Contact allergy in children continues to be underdiagnosed and should be studied, tested and publicized more often. The potential role of food chemicals as a possible cause of dermatitis and food allergy requires study and, conversely, exposure to shared haptens in cosmetics and food could potentially be a cause of food allergies [72]. Increasing exposure to palladium, with its high sensitizing potential, in electronic appliances, dental alloys, jewellery and car exhaust catalytic converters, presents a real risk of it becoming the ‘metal allergy of the 21^st^ century’ [73]. The epidemiology and dynamics of palladium sensitization call for an in depth study of palladium allergy from the scientific viewpoint and further regulation of its use.

Urticaria in all its presentations has an estimated lifetime prevalence of around 20% [74]. However, reliable epidemiologic data are scarce, and most of the published literature deals with adults. Thus, there is a need for epidemiological research into urticaria, as well as good diagnostic markers defining the subgroups within urticaria patients. In addition, treatment with new classes of drugs, including novel antihistamines, mast cell stabilizing drugs and biologicals, such as omalizumab [75], should be evaluated thoroughly for their clinical effectiveness and understanding the biology of mast cell disease. Development of specific mediator antagonists for hereditary angioedema and cryopyrin associated periodic fever syndrome will help to define the phenotype of specific subsets of disease.

### Environmental determinants of allergy

#### Aerobiology and pollution

Airborne allergens are a major trigger of allergic diseases, especially of the respiratory tract. Monitoring of biological airborne particles (pollen and moulds) is currently done by private networks. All over Europe, these national networks are underfunded and on the verge of collapse. In addition, European Union (EU)-citizens and scientists have limited access to pollen and mould data due to diffuse property rights, as most stations are funded with private money.

Therefore, there is an urgent need to secure, harmonize and open the existing networks with a European-scale pollen, allergen and air pollution/air quality forecasting network, also capable of predicting future developments in migration of allergenic plants (like *Ambrosia artemisiifolia*), and the environmental change in allergen exposure due to climate change. Thus, we will be able to sufficiently serve allergic sufferers and policy makers. The existing networks could be incorporated in the Global Monitoring for Environment and Security (GMES) Network for air quality monitoring and forecasting, in combination with the modeling communities.

#### Major unmet needs in aerobiology

• Harmonization of existing networks for monitoring biological airbourne particles

• Monitoring of the ‘exposome’ in conjunction with epidemiology in sensitization and symptom monitoring

• Identification of the socioeconomic burden of different biological airbourne particles to define strategies to fight pollution

• Identification of the role of climate change in allergic diseases

• Epigenetics studies to link the environmental factors with inherited inflammatory thresholds

The causes of allergic diseases are multifaceted. Thus, multiple exposure monitoring (‘exposome’) in conjunction with symptom and sensitization monitoring may reveal novel associations and interactions. Assessment of the ‘exposome’ (biological and chemical, indoor and outdoor) EU-wide, in concert with ongoing epidemiological studies/registries of allergic symptoms and sensitizations, will produce important synergies [76]. The climate change and its impact on development of allergies, microbiota, virus epidemics, parasites, moulds and food resources should be specifically focused on [77].

Pollen, moulds, parasites, viruses and bacteria are the carriers of allergens and/or innate immune system danger signals. These biological properties (not the pollen or the mould but the allergen and the danger signal) in ambient air are currently only partly monitored and should be an addition to current research. Cross border comparison, also with non-EU countries will enlarge the biological variance and thus facilitate the finding of allergy modulating factors [78,79].

### Infections and allergy

#### Major research gaps on the role of infections in allergy

• Differential immune response to various microorganisms

• Helminth infections as a paradigm to prevent/treat allergy

• Evolution and dynamics of the microbiome

• Viral-bacterial interactions

• Development of vaccination and other targeted interventions against human rhinoviruses

• Determine mechanisms underlying deficient antiviral immune responses in asthma

Both infections and commensal micro-organisms have a crucial role in the development and severity of allergic disorders and are involved in their resolution or chronicity. To harness their properties for prevention and treatment of allergies, we need to better understand which micro-organisms affect allergies naturally and how they interact with immune and inflammatory responses in allergy. This requires considerable epidemiological, clinical and mechanistic research both in human and animal models.

Helminth infections are associated with reduced skin prick test responsiveness to environmental allergens, especially if the parasite burden is high and occurs in early life. The evidence with regard to allergic disease is more heterogeneous, but some studies show a negative relationship between allergy and certain parasite infections, such as hookworm and schistosomiasis [80]. However, clinical trials treating people with established allergy with a single parasite dose have not shown beneficial effects so far. Future research will need to include trials of multi-dose administration resulting in higher worm burden and stronger immunomodulation. Another approach could be to develop allergy therapies and prophylaxis based on parasite-derived products; such products should be assessed for efficacy and safety, especially in young children, who are likely targets for primary prevention of allergy [81].

There is also emerging evidence that exposure to both commensal and pathogenic bacteria may influence the development of allergy [82]. We need to understand in detail how the microbiomes of gut, skin, and airways evolve from early life to senescence, which factors (e.g. birth mode, breastfeeding, diet, puberty, pregnancy, infections) influence their composition and how this relates to the development and severity of allergies. Such studies will also contribute to investigations aiming at the development of preventive and therapeutic applications of pre-, pro- and symbiotics.

During the last decades, it has become evident that viral infections, particularly those caused by human rhinoviruses (RV) are the most frequent triggers of acute exacerbations of asthma; in some cases viral agents have been detected in more than 90% of such events [83,84]. RVs are also responsible for the majority of mild rhinitis, i.e. common colds, therefore contributing further to symptoms in respiratory allergic patients. Viral bronchiolitis in infants is associated with an increased asthma risk. Once again RVs appear to be the agents most strongly associated, with a remarkable 40-fold increase of risk of wheezing at age 6 after an early symptomatic RV infection [85]. Whether early life episodes of respiratory viral infections also predispose to the induction of sensitization is less well-established, but has potentially great impact on strategies for allergy prevention. Given the lack of effective treatment or prevention of virus-induced asthma symptoms, future research must focus on developing diagnostic, therapeutic and prophylactic modalities for virus induced respiratory allergy. Our incomplete understanding of the role that respiratory viruses play in immune and inflammatory responses of the respiratory tract, their regulation and resolution, requires research in both animal models and in well-defined patient populations [86]. This needs to include studies to determine mechanisms underlying deficient antiviral immune responses reported in asthma [87] and define the roles of interactions between different viruses, including the recently identified rhinovirus-C [88], viral-bacterial synergistic effects, which accentuate airway inflammation [89], repeated infections, and interactions between viruses and other factors including allergens and pollutants, in establishing chronic respiratory allergies. Prospective cohort studies involving comprehensive microbiological detection and identification of other potential triggers of asthma development and exacerbations may be required to provide accurate information on natural microbial exposures and the relative impact of individual respiratory pathogens.

### Insect stings

Hymenoptera, which represent one of the largest orders of insects, are responsible for the majority of anaphylactic reactions in adults, of which some are fatal. In Europe, wasp or bee stings are the most common elicitors of Hymenoptera venom allergy (HVA) having a great impact on quality of life in adults, children and their parents [90]. Population-based studies should establish the regional frequency of HVA in different European countries [91]. Identification of the genetic background, and of the pathophysiological mechanisms involved in venom allergic reactions also require further research [92]. Healthcare providers and the public alike will benefit from proper diagnosis and long-term treatment of this potentially life-threatening allergy. To improve the diagnosis of HVA, certain techniques such as molecular diagnosis and peptide microarray immunoassay need further evaluation. A better knowledge on eliciting allergens might not only help to make a correct diagnosis but might also help to optimize venom extracts, or even replace them with recombinant allergens, for treatment. Parallel studies should aim at improving the clinical interpretation of well-established diagnostic procedures such as the intradermal tests, baseline serum tryptase concentration and sting challenge test [93].

Unlike food and medication allergy, which are managed primarily by allergen avoidance, the prospective management of HVA relies on venom immunotherapy (VIT), where treatment is mediated through administration of gradually increasing doses of the venom-allergen.

The efficacy of VIT in adults has progressed considerably, reaching a protective level of up to 98%. Still, there are many open questions regarding patient selection for VIT and duration of treatment. The long-term prognosis of untreated in comparison to treated HVA in children and adults needs further evaluation; only few data are available on risk factors of severe reactions in children with HVA [94]. Various treatment protocols mainly differ from each other with respect to the degree of tolerance of VIT, and it should be examined whether specific patients particularly profit from specific types of treatment protocols [95].

### Food, diet & nutrition

Food allergies affect a considerable proportion of children and adults in Europe and are the leading cause of severe anaphylaxis [96]. Furthermore, there is increased recognition of disorders associated with food allergy, such as eosinophilic oesophagitis.

Our current knowledge about the molecular mechanisms and factors for the allergic sensitization and elicitation phase is limited. Therefore, in depth characterization studies of both allergens and their co-factors are needed to understand how immune tolerance towards dietary antigens is disrupted and allergic sensitization initiated. An integrative approach is needed, that addresses the role of exposure (timing and dose) and host factors, including investigations on the role of genetic predisposition, the extent and composition of the intestinal microbiome and the involvement of the innate defense system [19,97].

In particular the interplay between the innate immune system and food should be studied in more detail. While we know that in spite of a close botanical relationship, peanut seems to be a strong sensitizer and soy a much weaker sensitizer, we still do not know whether the reason for this discrepancy is differences on the allergen level (including different digestibility), on the level of innate immune response stimulation, on the level of the different food matrices, on eating habits (oral exposure) or even on the level of non-oral exposures such as transdermal or inhalation. Thus both clinical studies of allergen exposure and uptake and *in vitro* models of the human intestine by which these features can be studied should be given high priority.

#### Unmet needs in food allergy

• Understand molecular mechanisms of tolerance and its disruption in food allergy

• Improve diagnosis with individual allergen components, food matrix interactions and threshold identification

• Establish food allergy phenotypes

• Establish novel immunotherapeutic approaches

• Evaluate the socioeconomic cost of food allergies

• Standardize nutritional interventions

For improved diagnosis of food allergy, complete panels of well characterized individual allergens and knowledge on their food matrix interactions regarding allergenicity, are still lacking. Identification of thresholds for each individual food is also needed. In component resolved diagnosis, investigation of allergen specific IgE recognition with clinical implications and the identification of marker allergens is needed at least for the most important food allergen sources. These well-characterized diagnostic tools will contribute to patient tailored dietary recommendations and to design avoidance strategies, accordingly; such approaches have been initiated within the context of the FP6-funded project EuroPrevall. On the other hand in depth knowledge of allergens and certified reference material available for the food industry will help them to fine tune their allergenic risk management. Furthermore, individual food allergens including their hypoallergenic variants will contribute to novel immunotherapeutic applications for food allergy.

Epidemiological and clinical studies suggest that there are different phenotypes in food allergy and that the onset (early, late), natural course (short term, persistent), and clinical presentation (mild, severe) differ. Multicentre studies involving a comprehensive clinical, immunological and (epi)genetic evaluation are needed to establish the different phenotypes in food allergy. This will further guide the management and specific treatment of food allergic patients.

Specific immunotherapy in food allergy, especially oral but also sublingual, has provided promising results. With the availability of hypoallergens, also subcutaneous immunotherapy has regained interest [98]. Studies ranging from basic science to clinical application are urgently needed regarding indications, underlying immunological mechanisms, safety, efficacy and cost-effectiveness of this treatment [98].

Recently, methods were developed to assess and record the reduced quality of life of the food allergic patient. However, the implementation of these findings in improved dietary recommendations, training of the patient or production of safer foods is still lacking and calls for a multidisciplinary approach to address the relevant issue. In parallel, the socioeconomic cost of food allergy is largely unknown. Capitalizing on a specific instrument built in the EuroPrevall project [99], the economic burden of food allergy can be studied across Europe, and the impact on costs of diagnostic and therapeutic interventions evaluated.

Despite the importance of diet and nutrition in immune development, only a small number of studies have evaluated their role on the prevention and natural course of food allergy and allergic diseases in general. There is therefore a need for descriptive studies on preventive nutritional aspects. These would include nutritional analysis around conception, during pregnancy, but also in early life and evaluation of atopic disease outcomes longitudinally. Nutritional analysis in lactating women as related to the composition of breast milk may also provide important insights. The role of specific nutrient levels, e.g. vitamin D, should be part of an extensive nutritional analysis [100].

The influence of the nutritional composition of avoidance diets on both nutritional status, but also disease severity, of children and adults with food allergy should be further evaluated [101].

Unlike other diagnostic tools, the removal of a suspected food from the diet has not been properly standardized nor validated. Furthermore, there is an important diagnostic value of a standardized allergy-focused diet history. Based on expert consensus opinion, both the recent NIH US guidelines and the UK NICE guidelines [102] reported that diet history is the cornerstone of food allergy diagnosis. Research efforts should gather evidence to substantiate this opinion, including the role of the dietitian in both diagnosis and individual dietary counseling.

Food labeling remains an important issue and EFA is actively involved in the elaboration and follow-up of the new EU regulation on food information to consumers.

### Drug allergies

Drug hypersensitivity reactions (DHRs) affect more than 7% of the general population and represent an important public health problem [103]. They are unpredictable, cause morbidity and mortality, compromise optimal medical care and are a major cause of post-marketing drug withdrawal [104]. While urticaria and maculopapular exanthemas are the most frequent, other drug-induced reactions, although less frequent may have a high fatality rate, including systemic anaphylaxis and toxic epidermal necrolysis (TEN).

DHRs represent a growing health problem and their world-wide prevalence is expected to increase over the next decades. A number of reasons will contribute to this phenomenon and will amplify its impact on the general population. First, new pharmacological molecules are continuously going to the market and more and more patients will be exposed to these agents. In particular, biological drugs, such as monoclonal antibodies, have profound interactions with the immune system and may cause severe hypersensitivity reactions. Second, there is an increasing use of over-the-counter medications that may potentially raise the risk of adverse reactions to drugs because of lack of medical supervision. Finally, the concomitant administration of multiple drugs (polychemotherapy), particularly in the elderly, is expected to increase the rate and severity of reactions to drugs: It is worth to mention, for example, that aspirin (ASA) and other drugs widely used for cardiovascular diseases (ACE inhibitors, beta-blockers) may exacerbate or aggravate allergic and anaphylactic reactions.

The complexity in DHRs is high; despite the common occurrence of DHRs, the mechanisms, diagnostic/therapeutic opportunities and differential diagnoses differ between drugs and manifestations and experience towards specific reactions is lacking. Every centre alone, experiences a limited and mostly biased spectrum of the disease and physicians frequently do not attempt to clarify a suspected reaction at all [105]. With diversity and diagnostic challenges, both individual patient phenotyping and epidemiological studies are affected. Additionally, most therapeutic recommendations, including new approaches such as desensitization are predominantly based on case reports or small case series. As we do not know the natural course of disease, it is not clear whether lifelong avoidance is really necessary. Taking into account that DHR research has not been supported for a long time by either the pharmaceutical industry or national programs [106], there is a clear need for training, standardized criteria, and large, multicentre studies and data collection to provide answers to the above questions. National and international registries should also be implemented or potentiated to provide useful information on the real dimension of the problem and to identify reliable parameters to assess the individual risk of DHRs.

New diagnostic tools have to be developed, as the available ones are poorly validated and may lead to misclassification (underreporting or overdiagnosis). The development of new procedures, such as standardization of skin tests, test concentrations, advancement and improvement of diagnostic test agents and tests, such as basophil activation test (BAT), Elispot, lymphocyte transformation test (LTT), is indispensable to confirm a DHR and identify the culprit drug to be avoided in the future. Existing diagnostic tests must be critically validated and standard operation procedures should be tailored to specific drugs, specific manifestations and age groups (children versus adults). Drug re-exposure by standardized challenges are most needed procedures in order to limit medication withdrawal based on an unclear diagnosis. Nevertheless, they are often non-standardized and lack good correlation with currently used diagnostic *in-vitro* tests.

#### Research agenda for drug hypersensitivity

• Training (multiple drugs, complex manifestations and mechanisms)

• Standardization of test procedures (still not achieved)

• Development of new diagnostic tools

• Reliable epidemiological data with proven hypersensitivity

• Clinical multicenter studies on diagnosis and therapy with well-phenotyped patients

• Mechanistic studies (pharmacological interaction with immune receptors (PI) concept, genetic pretesting, risk factors)

The availability of tissue and serum samples from immune-mediated drug hypersensitivity reaction (IDHR) patients is a prerequisite for basic research in the mechanism of DHRs, which may be allergic or non-allergic, with immunological or pharmacological recognition and with the allergenic determinants mostly unknown. In this effort, the study of genetic variants, especially of the major histocompatibility complex (MHC) molecules, should be included, in order to enable personalized recommendations for the risk to develop a DHR. To generate preclinical testing methods to assess the risk of potential DHRs in new drugs, research should also encompass the characterization of drug-specific (chemical structure, metabolites, exposure), intrinsic (genetics) and extrinsic risk factors (viral infections, other danger signals), complemented by preclinical prediction models.

On the socioeconomic level, as in other allergy manifestations, studies should define the impact of DHRs on the quality of life of patients and their cost on the health system.

### Activity-related allergies

#### Allergy and asthma in sports

A moderate physical activity favors health and well-being whereas a sedentary lifestyle and obesity are associated with a higher prevalence of allergic diseases in both children and adults [107]. Interestingly, however, an intense physical training, as a stress event, may induce several changes in immune parameters and response to environmental agents, which essentially result in an increased susceptibility to infections and in a preferential Th2 (allergic) phenotype [108].

#### Major research gaps on the role of allergy and asthma in sports

• Describe mechanisms of exercise-induced bronchoconstriction and identify biomarkers

• Understand the immune derangement after strenuous exercise

• Therapeutic trials in athletes

• Standardization of specific diagnostic tests

Physical exercise may also play a direct role on target organs triggering symptoms such as bronchial obstruction and anaphylaxis. Indeed, allergic diseases, asthma and exercise-induced bronchoconstriction (EIB) are present with high and increasing prevalence in athletes, significantly affecting their health status and performance and therefore representing a major concern in Sports Medicine [109]. The necessity therefore in Sports Allergy research is to describe the mechanisms of EIB, so as to clarify the clinical relevance and natural course of this phenotype and also identify markers of epithelial stress and inflammation. In this line of research, it would also be feasible to identify putative danger signals of immune derangement linked to the transient immune deficiency, which forms the basis for recurrent infections in elite athletes and is caused by strenuous and continuous training loads. Research efforts recruiting the –omics approaches should allow understanding of the impact of acute and chronic exercise as well as any correlation of gender on the immune functions.

With regard to clinical pharmacology in Sports Allergy, this is often extrapolated from data obtained in the general population, creating a need for randomized controlled clinical trials solely in athletes. Allergy and asthma in athletes is underdiagnosed [110] but this would be remedied if athletes were to be routinely evaluated through a standardized protocol for sensitization and immune function; such a policy would also allow improved management of their condition and permit the accurate study and screening of the effects on physical performance of anti-allergic and anti-asthmatic drugs (with special reference to beta-2 agonists). There is a need for guidelines for the diagnosis and management of allergic diseases and these should be widely diffused and applied by Sports physicians, in compliance with the World Anti-Doping Agency (WADA) rules, in the adequate management of these clinical pictures [111]. Some special attention should be paid to the usefulness of different methods of non-specific bronchial provocations in diagnosis of athletes’ asthma. Since metacholine provocation does not prove useful in a substantial percentage of cases, more comparative research involving other stimuli is needed in order to create feasible and unified algorithms to be used in professional athletes.

### Occupational allergy

#### Major research gaps in occupational allergy

• Changes of occupational allergy with time

• Public health impact of occupational allergy

• Monitor allergen exposure at workplaces

Occupational allergy and particularly occupational asthma can be caused by more than 300 agents and about 15% of adult-onset asthma can be attributed to workspace exposures [112,113]. Exposure-specific studies on occupational asthma have focused on substances of high and low molecular weight- e.g. flour, enzymes, isocyanates and latex [114]. While in developing countries the workforces probably have more extensive occupational exposures than in high-income countries, lower figures of the occurrence of occupational asthma have been reported, probably indicating a problem of underdetection and methodology. In addition, protective mechanisms described for respiratory allergies (hygiene hypothesis) may also play a role for occupational allergies. Still, the public health impact and burden to society related to occupational or work-related asthma have been scarcely investigated. Assessment of excess burden of disease due to specific occupational exposure is a useful measure, when there is valid information on population exposure and attributable fractions. Changes in the working conditions and implementation of new substances lead to the introduction of new allergens in some workplaces and the onset of new, so far unknown cases of occupational allergy. However, there is only little available information on the changes in the pattern of occupational allergy over time. An objective evaluation of the time trends in the incidence and causes of immunological occupational asthma in the EU, using workers' compensation data or registry-based data would be of great importance [115-118]. Prevention of occupational asthma related to a work-sensitizing agent would ideally be achieved by avoidance of exposure causing immunological sensitization and subsequent asthma. Especially apprenticeship is a period of increased risk of developing work-related respiratory allergic diseases and therefore is a need for appropriate professional advice to young adults aiming to reduce unsuitable job choices and prevent impairment from their careers [119]. Different forms and different steps of prevention exist, like primary prevention (the ideal form of prevention), where workers do not become sensitized to agents that can cause asthma. Since primary prevention is not always successful, secondary prevention by medical surveillance has some evidence to support benefit for those working with common occupational sensitizers. On the other hand it is necessary to optimize workers’ education and also the diagnosis and medical management to minimize further impairment of the airways [120-124]. There is a need to increase the implementation of airborne allergen quantification at workplaces to be used as scientific background e.g. for the discussion of health-based occupational exposure limits for high molecular weight sensitizers.

### Allergic diseases in children

Childhood is a key period as many allergies start early in life. It is also characterized by a large number of research needs, mainly due to the lack of reliable evidence to guide practice that has resulted from the challenges associated with undertaking paediatric research. We are still uncertain how to prevent children from developing allergic diseases. For example, important advances in our knowledge of genetic associations with allergic disease, have not clarified the underlying pathological pathways, probably because we have yet to understand their interactions with environmental exposures. We also lack knowledge on epigenetic mechanisms, now thought to be important in allergy. A relatively recent hypothesis in the prevention field is that high dose oral exposure to food allergens in early infancy may promote the development of immunological tolerance [125]. We must await the results of large, interventional studies to assess the validity of this hypothesis and it will be important to explore whether it also holds for aeroallergens, opening up possibilities for preventive vaccination. Additionally, with our improved understanding of the infant immune system, new therapeutic approaches addressing early immune stimulation and tolerance induction using bacterial compounds need further exploration [126].

Important gaps exist in the diagnostic tools that are available for young patients with possible allergy. In practice, it is not uncommon to find children with symptoms highly suggestive of clinical allergy, but negative allergy skin or blood tests. There is a need to investigate the potential importance of novel allergens and whether we can detect the local presence of relevant specific IgE in relevant end organs in addition to the skin or serum. Furthermore, our current allergy skin or blood tests fail to distinguish between clinical allergy and irrelevant sensitization or to identify patients at high risk of severe allergy mediated reactions [127]. We need a new improved approach to diagnostic testing to reduce the need for potentially dangerous provocations challenges (e.g. food challenges).

#### Research needs in paediatric allergy

• More studies in children of different ages are needed, both on the development of allergy and to evaluate differential responses to treatment

• Large, primary prevention intervention programs to confirm current hypotheses and reverse the epidemic

• Improved, non-invasive diagnostic tools

• Prevention and/or treatment of asthma exacerbations

• Evaluate the long-term effects of immunotherapy in children

• Focus on adolescence

Asthma is an important problem in childhood affecting many children and resulting in significant morbidity [128]. It has been suggested that vitamin D deficiency may play an important role in the development of asthma as well as being associated with severe asthma [129]. Like many findings in paediatric allergy, these are based on observational data that are very susceptible to confounding. There is a research need for multi-centre randomized controlled trials to assess the role of vitamin D in the development of asthma and/or severe asthma. Another unmet need in paediatric asthma is the study of exacerbations, many of which are associated with viral respiratory tract infections [83]; we also do not currently have any therapies capable of preventing or treating such exacerbations. A possible role of early respiratory virus exposure in facilitating sensitization to allergens requires attention as well. A further unexplained association exists between food allergy and severe exacerbations of asthma [130], which is important particularly as asthma is also associated with severe allergic reactions to foods. Severe asthma in childhood, and adult life, is poorly understood. In an attempt to understand this area, a novel unbiased “omics” systems biology approach is currently being used to explore the underlying pathophysiological mechanisms [131]. A similar approach is also being used in other allergic diseases and both studies can be expected to highlight many novel pathways that have the potential to deliver new therapeutic approaches for allergic disease.

The majority of our therapies for allergic disease only suppress the disease process while they are taken. The exception is immunotherapy, which would appear to have the potential to alter the natural history of allergic disease in childhood, for example preventing the development of asthma in children with hay fever [132]. Again though, as the quality of the paediatric evidence in this area is not optimal there is a need for well designed, double-blind, placebo-controlled studies to assess the long-term effects of immunotherapy in childhood. While such studies are ethically challenging, it is important to characterize the long-term effects, as this would substantially alter the perceived cost-effectiveness and trade-off between efficacy and safety of immunotherapy in childhood.

Adolescents and young adults are at higher than expected risk of morbidity and mortality from both asthma and food allergy. While this is poorly understood, it may result from the disengagement of adolescent patients from their medical needs as they are challenged by the usual process of adolescence [133]. It is also not assisted by the lack of continuity between paediatric and adult medical care in many countries. There is a need to develop a better, adolescent-focused approach to this at risk group to tackle this unmet need [134] and to develop better transition strategies to ease the progression from paediatric to adult services.

### Causal treatment of allergy: allergen specific immunotherapy

Allergen specific immunotherapy is the only currently available medical intervention that has the potential to affect the natural course of the disease [135]. Accumulating evidence have convincingly shown that in addition to alleviating symptoms, allergen specific immunotherapy can improve quality of life, reduce long-term costs and burden of allergies, and has the potential to change the course of the disease. Several appropriately designed and powered clinical trials have proven its good safety profile and effectiveness in allergic rhinitis, asthma and venom allergy [59,136,137].

However, there are still important questions to be answered. The unmet needs of this causal treatment should be evaluated in an integrated multinational academic, research, industry and regulatory agencies effort in Europe. Research should focus on optimizing clinical and immunological efficacy, safety and compliance, possibly through improved time-schedules. Novel approaches such as the use of adjuvants, modified allergens and molecular allergen components should be widely evaluated [138].

#### Major knowledge gaps in allergen-specific immunotherapy

• Mechanisms of induction and long term maintenance of allergen tolerance

• Molecular mechanisms of how T-reg cells and B-reg cells are generated in vivo and how to affect their life span

• Role of resident tissue cells in immune tolerance

• Molecular mechanisms of spontaneous healing, remissions and exacerbations of allergic disease

• Local tissue events during SLIT and epicutaneous SIT

• Better adjuvants that specifically induce immune tolerance

• Early biomarkers and predictors to decide to start, stop and success

• Phase III clinical trial primary outcomes equivalent to real-life exposure

• Efficient short term and long lasting treatment modalities

Outcome measures for both clinical trials and routine clinical practice should be further standardized and validated. To date, the required primary outcome is real-life exposure, which by its nature is very poorly standardized. Alternatives are urgently needed, not only because costs of failed trials due to lack of pollen exposure are a great financial burden to the sponsor, but perhaps even more importantly it is not ethical to subject patients to double-blind, placebo-controlled (DBPC) trials in which exposure is at best poorly controlled. Another issue is the dominance of poly-sensitization in clinical trials conducted for one allergy. Clear indications and guidelines, especially in regard to polysensitized patients are therefore needed. Multi-allergen immunotherapy needs more supporting data to be validated. The use of surrogate antigen challenges in the eye and nose and their relationship to clinical efficacy of SIT should be evaluated for both, seasonal and perennial allergens. The use of pollen chambers in dose–response studies has to be adequately studied. Surrogate allergen challenges should also be evaluated as an alternative primary outcome for phase III clinical trials. New modalities of allergen-applications such as the epicutaneous and the direct injection of allergens in the lymph node are promising and need to be further evaluated. The long-term efficacy and safety of these modalities needs to be confirmed.

In children, there is a need to identify an optimal dose, dosing frequency and duration. Most importantly however, long-term efficacy, including the preventive capacity for asthma and new sensitisations, and the safety profile of allergen specific immunotherapy should be further addressed in the paediatric population. This will facilitate the recognition and approval of allergen specific immunotherapy in children by regulatory national and international agencies. The demonstration of long-term efficacy is required for the current mandatory paediatric investigation plan (PIP) that must accompany applications for marketing authorization submitted to the European Medicine Agency (EMA). In addition, a role of SIT for secondary prevention in already sensitized children has been evoked, but requires further evaluation. Studies exploring this hypothesis are definitely needed. Furthermore, there is a need to perform economic assessments in high-quality prospective and long-term clinical studies comparing immunotherapy with pharmacotherapy in real-life practice.

Understanding the mechanisms of immunotherapy will ultimately lead to advanced, curative treatments of allergic diseases, therefore research focusing on the generation, life span and different roles of T-regulatory cells is needed [34,139]. Furthermore, long-term immune tolerance and local tissue events should be studied in detail, in parallel to the understanding of spontaneous healing, remission and exacerbations of allergic disease. Specific issues in relation to immune tolerance include the molecular mechanisms of how T regulatory cells are generated in vivo, their life span induced by allergen immunotherapy, whether they have any deleterious roles, tolerance to tumour antigens and chronic infectious agents and the role of resident tissue cells [140]. Increased knowledge on tolerance induction may also lead to strategies for preventive vaccination [141].

Biomarkers able to predict clinical response, treatment outcome and/or monitor progression of the treatment are also key to enhance clinical decisions. Any differences in the mechanisms of high-dose and low-dose allergen specific immunotherapy need also to be assessed.

## Conclusions

The advances in allergy diagnosis, management and basic science to date have been substantial. There remain, nevertheless, many unmet needs as a consequence of the modern epidemic of allergy. Extensive research is needed to counteract the consequences for both individual patients and public health. Despite the variety of external factors that may trigger allergic reactions, as well as the pathophysiological complexity of these chronic diseases, common themes in relation to research needs arise: phenotyping/endotyping seems to be widely required in order to arrange or re-arrange clinical syndromes in more coherent and treatment-responsive groups. Using different –omics approaches in combination with systems biology and systems medicine appear promising in this direction. Using these approaches, patient-tailored management, including gender specific focusing, may become realistic. The need for epidemiological description or surveillance, including exposome monitoring, remains in many fields, and real-life, patient-centered research supported by registries is required. Basic mechanisms are still incompletely understood, therefore, any diagnostic, classification or treatment effort should be supported by attempts to better understand pathophysiology. Allergen components promise to be the next generation of diagnostic and therapeutic tools; large-scale studies are required to bring them to the clinics. Finally, severe disease, co-morbidities and/or exacerbations over a chronic course, are usually the most difficult to manage, more costly and dangerous and therefore require particular attention. Nevertheless, currently arising treatments, particularly in the fields of immunotherapy and biologicals, hold great promise for targeted and causal management of allergic diseases.

### Major research themes in allergy

• Mechanisms

• Prevention

• Epidemiological surveillance - Registries

• Biomarkers for prediction, diagnosis, classification, treatment response

• Phenotypes / Endotypes

• Severe Disease/Exacerbations

• Novel treatments (biologicals, vaccines, new drugs)

## Abbreviations

AD: Atopic dermatitis; AKC: Atopic keratoconjunctivitis; AR: Allergic rhinitis; DBPC: Double-blind, placebo-controlled; DHR: Drug hypersensitivity reaction; EAACI: European Academy of Allergy and Clinical Immunology; ECHRS: European community respiratory health survey; EIB: Exercise-induced bronchoconstriction; EU: European Union; GA^2^LEN: Global Allergy and Asthma European Network; HVA: Hymenoptera venom allergy; IDHR: Immune-mediated drug hypersensitivity reaction; ILCs: Innate lymphoid cells; ISAAC: International study of asthma and allergies in childhood; LTi: Lymphoid tissue–inducer; PAC: Perennial allergic conjunctivitis; VIT: Venom immunotherapy; VKC: Vernal keratoconjunctivitis; WADA: World anti-doping agency.

## **Competing interests**

All authors are members of EAACI or EFA Boards and declare no other competing interests in respect to this publication.

## References

[B1] World Health Organization FSn307 http://www.who.int/mediacentre/factsheets/fs307/en/index.html

[B2] Asher MI Montefort S Bjorksten B Lai CK Strachan DP Weiland SK Williams H Worldwide time trends in the prevalence of symptoms of asthma, allergic rhinoconjunctivitis, and eczema in childhood: ISAAC Phases One and Three repeat multicountry cross-sectional surveys Lancet 2006 368 9537 733 743 10.1016/S0140-6736(06)69283-0 16935684

[B3] European Federation of Allergy and Airway diseases, Patients Associations, (EFA) Fighting for breath http://wwwefanetorg/activities/documents/Fighting_For_Breath1pdf

[B4] Jarvis D Burney P ABC of allergies. The epidemiology of allergic disease BMJ 1998 316 7131 607 610 10.1136/bmj.316.7131.607 9518918 PMC1112638

[B5] European Community Respiratory Health Survey Variations in the prevalence of respiratory symptoms, selfreported asthma attacks and the use of asthma medication in the European Community respiratory health survey (ECRHS) Eur Respir J 1996 9 687 695 8726932 10.1183/09031936.96.09040687

[B6] E V EFA Book on Respiratory Allergies – Raise Awareness, Relieve the Burden 2011 http://wwwefanetorg/documents/EFABookonRespiratoryAllergiesFINALpdf

[B7] Wertz DA Pollack M Rodgers K Bohn RL Sacco P Sullivan SD Impact of asthma control on sleep, attendance at work, normal activities, and disease burden Ann Allergy Asthma Immunol 2010 105 2 118 123 10.1016/j.anai.2010.05.009 20674821

[B8] European Respiratory Society, [ERS] European Lung Book. The First Comprehensive Survey on Respiratory Health in Europe

[B9] Foundation EL Economic Impact of Lung Diseases 2011

[B10] Verboom P Hakkaart-Van L Sturkenboom M De Zeeuw R Menke H Rutten F The cost of atopic dermatitis in the Netherlands: an international comparison Br J Dermatol 2002 147 4 716 724 10.1046/j.1365-2133.2002.04964.x 12366418

[B11] Mancini AJ Kaulback K Chamlin SL The socioeconomic impact of atopic dermatitis in the United States: a systematic review Pediatr Dermatol 2008 25 1 1 6 10.1111/j.1525-1470.2007.00572.x 18304144

[B12] Council of the European Union Council conclusions on prevention, early diagnosis and treatment of chronic respiratory diseases in children http://wwwconsiliumeuropaeu/uedocs/cms_Data/docs/pressdata/en/lsa/126522pdf

[B13] Kauppi P Linna M Martikainen J Makela MJ Haahtela T Follow-up of the Finnish Asthma Programme 2000–2010: reduction of hospital burden needs risk group rethinking Thorax 2012 [Epub ahead of print] PMID 22504963 10.1136/thoraxjnl-2011-201028 22504963

[B14] Beasley RKU von Mutius E Pearce N International Study of Asthma and Allergies in Childhood (ISAAC) Steering Committee Worldwide variation in prevalence of symptoms of asthma, allergic rhinoconjunctivitis, and atopic eczema: ISAAC Lancet 1998 351 1225 1232 10.1016/S0140-6736(97)07302-9 9643741

[B15] Sunyer J Anto JM Tobias A Burney P Generational increase of self-reported first attack of asthma in fifteen industrialized countries. European Community Respiratory Health Study (ECRHS) Eur Respir J 1999 14 4 885 891 10.1034/j.1399-3003.1999.14d26.x 10573237

[B16] Bousquet J Kauffmann F Demoly P Leynaert B Bousquet PJ Demenais F Lenzen G Burney PG Zuberbier T Van Cauwenberge P [GA2LEN (Global Allergy and Asthma European Network)] Rev Mal Respir 2009 26 6 577 586 10.1016/S0761-8425(09)74689-3 19623103

[B17] Burney P Summers C Chinn S Hooper R van Ree R Lidholm J Prevalence and distribution of sensitization to foods in the European Community Respiratory Health Survey: a EuroPrevall analysis Allergy 2010 65 9 1182 1188 20180791 10.1111/j.1398-9995.2010.02346.x

[B18] von Mutius E Vercelli D Farm living: effects on childhood asthma and allergy Nat Rev Immunol 2010 10 12 861 868 10.1038/nri2871 21060319

[B19] Brown SJ Asai Y Cordell HJ Campbell LE Zhao Y Liao H Northstone K Henderson J Alizadehfar R Ben-Shoshan M Loss-of-function variants in the filaggrin gene are a significant risk factor for peanut allergy J Allergy Clin Immunol 2011 127 3 661 667 10.1016/j.jaci.2011.01.031 21377035 PMC3081065

[B20] Akdis CA Akdis M Trautmann A Blaser K Immune regulation in atopic dermatitis Curr Opin Immunol 2000 12 641 646 10.1016/S0952-7915(00)00156-4 11102766

[B21] Larche M Akdis CA Valenta R Immunological mechanisms of allergen-specific immunotherapy Nat Rev Immunol 2006 6 10 761 771 10.1038/nri1934 16998509

[B22] Gilles S Fekete A Zhang X Beck I Blume C Ring J Schmidt-Weber C Behrendt H Schmitt-Kopplin P Traidl-Hoffmann C Pollen metabolome analysis reveals adenosine as a major regulator of dendritic cell-primed T(H) cell responses J Allergy Clin Immunol 2011 127 2 454 461 10.1016/j.jaci.2010.12.1082 21281872

[B23] Basinski TM Holzmann D Eiwegger T Zimmermann M Klunker S Meyer N Schmid-Grendelmeier P Jutel M Akdis CA Dual nature of T cell-epithelium interaction in chronic rhinosinusitis J Allergy Clin Immunol 2009 124 1 74 80 e71-78 10.1016/j.jaci.2009.04.019 19523671

[B24] Akdis CA Allergy and hypersensitivity: mechanisms of allergic disease Curr Opin Immunol 2006 18 6 718 726 10.1016/j.coi.2006.09.016 17029937

[B25] Hammad H Chieppa M Perros F Willart MA Germain RN Lambrecht BN House dust mite allergen induces asthma via Toll-like receptor 4 triggering of airway structural cells Nat Med 2009 15 4 410 416 10.1038/nm.1946 19330007 PMC2789255

[B26] Mjosberg JM Trifari S Crellin NK Peters CP van Drunen CM Piet B Fokkens WJ Cupedo T Spits H Human IL-25- and IL-33-responsive type 2 innate lymphoid cells are defined by expression of CRTH2 and CD161 Nat Immunol 2011 12 11 1055 1062 10.1038/ni.2104 21909091

[B27] Strid J Sobolev O Zafirova B Polic B Hayday A The intraepithelial T cell response to NKG2D-ligands links lymphoid stress surveillance to atopy Science 2011 334 6060 1293 1297 10.1126/science.1211250 22144628 PMC3842529

[B28] Kau AL Ahern PP Griffin NW Goodman AL Gordon JI Human nutrition, the gut microbiome and the immune system Nature 2011 474 7351 327 336 10.1038/nature10213 21677749 PMC3298082

[B29] Ashe A Sapetschnig A Weick EM Mitchell J Bagijn MP Cording AC Doebley AL Goldstein LD Lehrbach NJ Le Pen J PiRNAs Can Trigger a Multigenerational Epigenetic Memory in the Germline of C. elegans Cell 2012 150 1 88 99 10.1016/j.cell.2012.06.018 22738725 PMC3464430

[B30] Eyerich S Eyerich K Pennino D Carbone T Nasorri F Pallotta S Cianfarani F Odorisio T Traidl-Hoffmann C Behrendt H Th22 cells represent a distinct human T cell subset involved in epidermal immunity and remodeling J Clin Invest 2009 119 12 3573 3585 19920355 10.1172/JCI40202 PMC2786807

[B31] Burgler S Ouaked N Bassin C Basinski TM Mantel PY Siegmund K Meyer N Akdis CA Schmidt-Weber CB Differentiation and functional analysis of human T(H)17 cells J Allergy Clin Immunol 2009 123 3 588 595 10.1016/j.jaci.2008.12.017 19178935

[B32] Stassen M Schmitt E Bopp T From interleukin-9 to T helper 9 cells Ann N Y Acad Sci 2012 1247 56 68 10.1111/j.1749-6632.2011.06351.x 22235761

[B33] Wisniewski JA Borish L Novel cytokines and cytokine-producing T cells in allergic disorders Allergy Asthma Proc 2011 32 2 83 94 10.2500/aap.2011.32.3428 21439160

[B34] Akdis CA Therapies for allergic inflammation: refining strategies to induce tolerance Nat Med 2012 18 5 736 749 10.1038/nm.2754 22561837

[B35] Pelaia G Renda T Romeo P Busceti MT Maselli R Omalizumab in the treatment of severe asthma: efficacy and current problems Ther Adv Respir Dis 2008 2 6 409 421 10.1177/1753465808100431 19124386

[B36] Wenzel SE Eosinophils in asthma--closing the loop or opening the door? N Engl J Med 2009 360 10.1056/NEJMe0900334 19264692

[B37] Eberlein B Krischan L Darsow U Ollert M Ring J Double positivity to bee and wasp venom: Improved diagnostic procedure by recombinant allergen-based IgE testing and basophil activation test including data about cross-reactive carbohydrate determinants J Allergy Clin Immunol 2012 14 14 10.1016/j.jaci.2012.02.008 22421265

[B38] Harwanegg C Hiller R Protein microarrays in diagnosing IgE-mediated diseases: spotting allergy at the molecular level Expert Rev Mol Diagn 2004 4 4 539 548 10.1586/14737159.4.4.539 15225101

[B39] Scala E Alessandri C Bernardi ML Ferrara R Palazzo P Pomponi D Quaratino D Rasi C Zaffiro A Zennaro D Cross-sectional survey on immunoglobulin E reactivity in 23,077 subjects using an allergenic molecule-based microarray detection system Clin Exp Allergy 2010 40 6 911 921 10.1111/j.1365-2222.2010.03470.x 20214663

[B40] Hoffmann HJ Bogebjerg M Nielsen LP Dahl R Lysis with Saponin improves detection of the response through CD203c and CD63 in the basophil activation test after crosslinking of the high affinity IgE receptor FcepsilonRI Clin Mol Allergy 2005 3 10 10.1186/1476-7961-3-10 15996266 PMC1201566

[B41] Ebo DG Bridts CH Hagendorens MM Mertens CH De Clerck LS Stevens WJ Flow-assisted diagnostic management of anaphylaxis from rocuronium bromide Allergy 2006 61 8 935 939 10.1111/j.1398-9995.2006.01094.x 16867045

[B42] El-Ghaiesh S Monshi MM Whitaker P Jenkins R Meng X Farrell J Elsheikh A Peckham D French N Pirmohamed M Characterization of the antigen specificity of T-cell clones from piperacillin-hypersensitive patients with cystic fibrosis J Pharmacol Exp Ther 2012 341 3 597 610 10.1124/jpet.111.190900 22371438 PMC3362878

[B43] Patrick DL Burke LB Powers JH Scott JA Rock EP Dawisha S O'Neill R Kennedy DL Patient-reported outcomes to support medical product labeling claims: FDA perspective Value Health 2007 10 Suppl 2 S125 137 17995471 10.1111/j.1524-4733.2007.00275.x

[B44] Baiardini I Bousquet PJ Brzoza Z Canonica GW Compalati E Fiocchi A Fokkens W van Wijk RG La Grutta S Lombardi C Recommendations for assessing patient-reported outcomes and health-related quality of life in clinical trials on allergy: a GA(2)LEN taskforce position paper Allergy 2010 65 3 290 295 10.1111/j.1398-9995.2009.02263.x 19930232

[B45] European Medicines Agency, Committee for medicinal products for human use, (CHMP) Reflection paper on the regulatory guidance for the use of health-related quality of life (HROL) measures in the evaluation of medicinal products European Medicines Agency website 2005 (cited 2007 April) 2005

[B46] US Department of Health and Human Services FDA Center for Drug Evaluation and Research Guidance for Industry: patient reported outcome measures: use in medical product development to support labelling claims: draft guidance Health Qual Life Outcomes 2006 4 79 17034633 10.1186/1477-7525-4-79 PMC1629006

[B47] Guyatt GH Oxman AD Vist GE Kunz R Falck-Ytter Y Alonso-Coello P Schunemann HJ GRADE: an emerging consensus on rating quality of evidence and strength of recommendations BMJ 2008 336 924 926 10.1136/bmj.39489.470347.AD 18436948 PMC2335261

[B48] Leynaert B Soussan D Monitoring the quality-of-life in allergic disorders Curr Opin Allergy Clin Immunol 2003 3 3 177 183 10.1097/00130832-200306000-00005 12840700

[B49] Baiardini I Braido F Brandi S Tarantini F Bonini S Bousquet PJ Zuberbier T Demoly P Canonica GW The impact of GINA suggested drugs for the treatment of asthma on Health-Related Quality of Life: a GA(2)LEN review Allergy 2008 63 8 1015 1030 10.1111/j.1398-9995.2008.01823.x 18691305

[B50] Baiardini I Braido F Tarantini F Porcu A Bonini S Bousquet PJ Zuberbier T Demoly P Canonica GW ARIA-suggested drugs for allergic rhinitis: what impact on quality of life? A GA2LEN review Allergy 2008 63 6 660 669 10.1111/j.1398-9995.2008.01649.x 18445183

[B51] van derVelde JL Flokstra-de Blok BM Vlieg-Boerstra BJ Oude Elberink JN Schouten JP Dunngalvin A Hourihane JO Duiverman EJ Dubois AE Test-retest reliability of the Food Allergy Quality of Life Questionnaires (FAQLQ) for children, adolescents and adults Qual Life Res: Inst J Qual Life Aspects Treatment, Care Rehabil 2009 18 2 245 251 10.1007/s11136-008-9434-2 19142745

[B52] World Health Organization Fact Sheet n°307 2011 http://www.who.int/mediacentre/factsheets/fs307/en/index.html

[B53] Holgate S Bisgaard H Bjermer L Haahtela T Haughney J Horne R McIvor A Palkonen S Price DB Thomas M The Brussels Declaration: the need for change in asthma management Eur Respir J 2008 32 6 1433 1442 10.1183/09031936.00053108 19043008

[B54] Lane S Molina J Plusa T An international observational prospective study to determine the cost of asthma exacerbations (COAX) Respir Med 2006 100 3 434 450 10.1016/j.rmed.2005.06.012 16099149

[B55] Sears MR Greene JM Willan AR Wiecek EM Taylor DR Flannery EM Cowan JO Herbison GP Silva PA Poulton R A longitudinal, population-based, cohort study of childhood asthma followed to adulthood N Engl J Med 2003 349 15 1414 1422 10.1056/NEJMoa022363 14534334

[B56] Lotvall J Akdis CA Bacharier LB Bjermer L Casale TB Custovic A Lemanske RF Jr Wardlaw AJ Wenzel SE Greenberger PA Asthma endotypes: a new approach to classification of disease entities within the asthma syndrome J Allergy Clin Immunol 2011 127 2 355 360 10.1016/j.jaci.2010.11.037 21281866

[B57] Agache I Akdis C Jutel M Virchow JC Untangling asthma phenotypes and endotypes Allergy 2012 67 7 835 846 10.1111/j.1398-9995.2012.02832.x 22594878

[B58] Auffray C Charron D Hood L Predictive, preventive, personalized and participatory medicine: back to the future Genome Med 2010 2 8 57 10.1186/gm178 20804580 PMC2945014

[B59] Greiner AN Hellings PW Rotiroti G Scadding GK Allergic rhinitis Lancet 2011 378 9809 2112 2122 10.1016/S0140-6736(11)60130-X 21783242

[B60] Maurer M Zuberbier T Undertreatment of rhinitis symptoms in Europe: findings from a cross-sectional questionnaire survey Allergy 2007 62 9 1057 1063 10.1111/j.1398-9995.2007.01367.x 17581263

[B61] Bousquet J Bachert C Canonica GW Mullol J Van Cauwenberge P Jensen CB Fokkens WJ Ring J Keith P Gopalan G Efficacy of desloratadine in persistent allergic rhinitis - a GA(2)LEN study Int Arch Allergy Immunol 2010 153 4 395 402 10.1159/000316351 20559006

[B62] Watelet JB Van Zele T Gjomarkaj M Canonica GW Dahlen SE Fokkens W Lund VJ Scadding GK Mullol J Papadopoulos N Tissue remodelling in upper airways: where is the link with lower airway remodelling? Allergy 2006 61 11 1249 1258 10.1111/j.1398-9995.2006.01226.x 17002699

[B63] Scadding G Hellings P Alobid I Bachert C Fokkens W van Wijk RG Gevaert P Guilemany J Kalogjera L Lund V Diagnostic tools in Rhinology EAACI position paper Clin Transl Allergy 2011 1 1 2 10.1186/2045-7022-1-2 22410181 PMC3294630

[B64] Hellings PW Dobbels F Denhaerynck K Piessens M Ceuppens JL De Geest S Explorative study on patients' perceived knowledge level, expectations, preferences and fear for side effects for treatment for allergic rhinitis Clin Transl Allergy 2012 2 1 9 10.1186/2045-7022-2-9 22643067 PMC3447732

[B65] Kong HH OJ Deming C Conlan S Grice EA Beatson MA Nomicos E Polley EC Komarow HD Murray PR Turner ML Segre JA NISC Comparative Sequence Program Temporal shifts in the skin microbiome associated with disease flares and treatment in children with atopic dermatitis Genome Res 2012 Epub ahead of print 10.1101/gr.131029.111 PMC3337431 22310478

[B66] Fox ATSP du Toit G Syed H Lack G Household peanut consumption as a risk factor for the development of peanut allergy J Allergy Clin Immunol 2009 123 2 417 423 10.1016/j.jaci.2008.12.014 19203660

[B67] Schultz Larsen F Diepgen T Svensson A The occurrence of atopic dermatitis in north Europe: an international questionnaire study J Am Acad Dermatol 1996 34 5 Pt 1 760 764 8632070 10.1016/s0190-9622(96)90009-2

[B68] Williams H Robertson C Stewart A Ait-Khaled N Anabwani G Anderson R Asher I Beasley R Bjorksten B Burr M Worldwide variations in the prevalence of symptoms of atopic eczema in the international study of asthma and allergies in childhood J Allergy Clin Immunol 1999 103 1 Pt 1 125 138 9893196 10.1016/s0091-6749(99)70536-1

[B69] Kawasaki HNK Kubo A Hata T Shimizu A Mizuno H Yamada T Amagai M Altered stratum corneum barrier and enhanced percutaneous immune responses in filaggrin-null mice J Allergy Clin Immunol 2012 10.1016/j.jaci.2012.01.068 22409988

[B70] Soyka MB Wawrzyniak P Eiwegger T Holzmann D Treis A Wanke K Kast JI Akdis CA Defective epithelial barrier in chronic rhinosinusitis: The regulation of tight junctions by IFN-gamma and IL-4 J Allergy Clin Immunol 2012 10.1016/j.jaci.2012.05.052 22840853

[B71] Rantanen T The cause of the Chinese sofa/chair dermatitis epidemic is likely to be contact allergy to dimethylfumarate, a novel potent contact sensitizer Br J Dermatol 2008 159 1 218 221 10.1111/j.1365-2133.2008.08622.x 18503603

[B72] Spiewak R Food-provoked eczema: a hypothesis on the possible role of systemic contact allergy to haptens present in both cosmetics and foods Estetol Med Kosmetol 2011 1 1 35 40

[B73] Faurschou AMT Johansen JD Thyssen JP Metal allergen of the 21st Century - a review on exposure, epidemiology and clinical manifestations of palladium allergy95 Contact Dermatitis 2011 644 185 195 21392026 10.1111/j.1600-0536.2011.01878.x

[B74] Hellgren L The prevalence of urticaria in the total population Acta Allergol 1972 27 3 236 240 4678809 10.1111/j.1398-9995.1972.tb01420.x

[B75] Saini SRK Hsieh HJ Wong DA Conner E Kaplan A Spector S Maurer M A randomized, placebo-controlled, dose-ranging study of single-dose omalizumab in patients with H1-antihistamine-refractory chronic idiopathic urticaria J Allergy Clin Immunol 2011 128 3 567 573 10.1016/j.jaci.2011.06.010 21762974

[B76] Peden D Reed CE Environmental and occupational allergies J Allergy Clin Immunol 2010 125 2 Suppl 2 S150 160 20176257 10.1016/j.jaci.2009.10.073

[B77] Cecchi L D’Amato G Ayres JG Galan C Forastiere F Forsberg B Gerritsen J Nunes C Behrendt H Akdis C Projections of the effects of climate change on allergic asthma: the contribution of aerobiology Allergy 2010 65 9 1073 1081 20560904 10.1111/j.1398-9995.2010.02423.x

[B78] Buters JT Kasche A Weichenmeier I Schober W Klaus S Traidl-Hoffmann C Menzel A Huss-Marp J Kramer U Behrendt H Year-to-year variation in release of Bet v 1 allergen from birch pollen: evidence for geographical differences between West and South Germany Int Arch Allergy Immunol 2008 145 2 122 130 10.1159/000108137 17848805

[B79] Laatikainen T von Hertzen L Koskinen JP Makela MJ Jousilahti P Kosunen TU Vlasoff T Ahlstrom M Vartiainen E Haahtela T Allergy gap between Finnish and Russian Karelia on increase Allergy 2011 66 7 886 892 10.1111/j.1398-9995.2010.02533.x 21255037

[B80] Flohr C Quinnell RJ Britton J Do helminth parasites protect against atopy and allergic disease? Clin Exp Allergy 2009 39 1 20 32 10.1111/j.1365-2222.2008.03134.x 19128351

[B81] Erb KJ Can helminths or helminth-derived products be used in humans to prevent or treat allergic diseases? Trends Immunol 2009 30 2 75 82 10.1016/j.it.2008.11.005 19138565

[B82] Bisgaard H Li N Bonnelykke K Chawes BL Skov T Paludan-Muller G Stokholm J Smith B Krogfelt KA Reduced diversity of the intestinal microbiota during infancy is associated with increased risk of allergic disease at school age J Allergy Clin Immunol 2011 128 3 646 652 10.1016/j.jaci.2011.04.060 21782228

[B83] Papadopoulos NG Christodoulou I Rohde G Agache I Almqvist C Bruno A Bonini S Bont L Bossios A Bousquet J Viruses and bacteria in acute asthma exacerbations–a GA(2) LEN-DARE systematic review Allergy 2011 66 4 458 468 10.1111/j.1398-9995.2010.02505.x 21087215 PMC7159474

[B84] Skevaki CL Psarras S Volonaki E Pratsinis H Spyridaki IS Gaga M Georgiou V Vittorakis S Telcian AG Maggina P Rhinovirus-induced basic fibroblast growth factor release mediates airway remodeling features Clinical and translational allergy 2012 2 1 14 10.1186/2045-7022-2-14 22908984 PMC3492082

[B85] Jackson DJ Gangnon RE Evans MD Roberg KA Anderson EL Pappas TE Printz MC Lee WM Shult PA Reisdorf E Wheezing rhinovirus illnesses in early life predict asthma development in high-risk children Am J Respir Crit Care Med 2008 178 7 667 672 10.1164/rccm.200802-309OC 18565953 PMC2556448

[B86] Papadopoulos NG Xepapadaki P Mallia P Brusselle G Watelet JB Xatzipsalti M Foteinos G Van Drunen CM Fokkens WJ D’Ambrosio C Mechanisms of virus-induced asthma exacerbations: State-of-the-art. A GA2LEN and InterAirways document Allergy: European J Allergy Clin Immunol 2007 62 5 457 470 10.1111/j.1398-9995.2007.01341.x PMC7159480 17324199

[B87] Contoli M Message SD Laza-Stanca V Edwards MR Wark PA Bartlett NW Kebadze T Mallia P Stanciu LA Parker HL Role of deficient type III interferon-lambda production in asthma exacerbations Nat Med 2006 12 9 1023 1026 10.1038/nm1462 16906156

[B88] Miller EK Edwards KM Weinberg GA Iwane MK Griffin MR Hall CB Zhu Y Szilagyi PG Morin LL Heil LH A novel group of rhinoviruses is associated with asthma hospitalizations J Allergy Clin Immunol 2009 123 1 98 104 10.1016/j.jaci.2008.10.007 19027147 PMC7112283

[B89] Sun K Metzger DW Inhibition of pulmonary antibacterial defense by interferon-gamma during recovery from influenza infection Nat Med 2008 14 5 558 564 10.1038/nm1765 18438414

[B90] Oude Elberink J Venom immunotherapy (VIT): clinical efficacy and improvement in quality of life Drugs Today 2008 44 43 45 19221618

[B91] Bilo BM Bonifazi F Advances in hymenoptera venom immunotherapy Curr Opin Allergy Clin Immunol 2007 7 6 567 573 10.1097/ACI.0b013e3282f1eca5 17989536

[B92] Niedoszytko M Bruinenberg M de Monchy J Weersma RK Wijmenga C Jassem E Elberink JN Changes in gene expression caused by insect venom immunotherapy responsible for the long-term protection of insect venom-allergic patients Ann Allergy Asthma Immunol 2011 106 6 502 510 10.1016/j.anai.2011.01.007 21624750

[B93] Borer-Reinhold M Haeberli G Bitzenhofer M Jandus P Hausmann O Fricker M Helbling A Muller U An increase in serum tryptase even below 11.4 ng/mL may indicate a mast cell-mediated hypersensitivity reaction: a prospective study in Hymenoptera venom allergic patients Clin Exp Allergy 2011 41 12 1777 1783 10.1111/j.1365-2222.2011.03848.x 22092437

[B94] Rueff F Przybilla B Bilo MB Muller U Scheipl F Aberer W Birnbaum J Bodzenta-Lukaszyk A Bonifazi F Bucher C Predictors of side effects during the buildup phase of venom immunotherapy for Hymenoptera venom allergy: the importance of baseline serum tryptase J Allergy Clin Immunol 2010 126 1 105 111 10.1016/j.jaci.2010.04.025 20542320

[B95] Bilo MB Cinti B Brianzoni MF Braschi MC Bonifazi M Antonicelli L Honeybee venom immunotherapy: a comparative study using purified and nonpurified aqueous extracts in patients with normal Basal serum tryptase concentrations J Allergy 2012 869243 12 Epub 2012 Jan 12 10.1155/2012/869243 PMC3263618 22287975

[B96] Scurlock AM Vickery BP Hourihane JO Burks AW Pediatric food allergy and mucosal tolerance Mucosal Immunol 2010 3 4 345 354 10.1038/mi.2010.21 20505663

[B97] Fernandez-Rivas M Bolhaar S Gonzalez-Mancebo E Asero R van Leeuwen A Bohle B Ma Y Ebner C Rigby N Sancho AI Apple allergy across Europe: how allergen sensitization profiles determine the clinical expression of allergies to plant foods J Allergy Clin Immunol 2006 118 2 481 488 10.1016/j.jaci.2006.05.012 16890775

[B98] Zuidmeer-Jongejan L Fernandez-Rivas M Poulsen LK Neubauer A Asturias J Blom L Boye J Bindslev-Jensen C Clausen M Ferrara R FAST: Towards safe and effective subcutaneous immunotherapy of persistent life-threatening food allergies Clinical and translational allergy 2012 2 1 5 10.1186/2045-7022-2-5 22409908 PMC3386014

[B99] Mills EN Mackie AR Burney P Beyer K Frewer L Madsen C Botjes E Crevel RW van Ree R The prevalence, cost and basis of food allergy across Europe Allergy 2007 62 7 717 722 10.1111/j.1398-9995.2007.01425.x 17573717

[B100] Vassallo MF Camargo CA Jr Potential mechanisms for the hypothesized link between sunshine, vitamin D, and food allergy in children J Allergy Clin Immunol 2010 126 2 217 222 10.1016/j.jaci.2010.06.011 20624647

[B101] Noimark L Cox HE Nutritional problems related to food allergy in childhood Pediatr Allergy Immunol 2008 19 2 188 195 10.1111/j.1399-3038.2007.00700.x 18257908

[B102] Excellence NIfHaC NICE Clinical Guideline 116. Food Allergy in Children and Young People. Diagnosis and assessment of food allergy in children and young people in primary care and community settings 2011 http://guidance.nice.org.uk/CG116 10.3399/bjgp11X583498 PMC3123497 21722479

[B103] Gomes ECM Praca F Gomes L Marino E Demoly P Self-reported drug allergy in a general adult Portuguese population Clin Exp Allergy 2004 34 1597 1601 10.1111/j.1365-2222.2004.02070.x 15479276

[B104] Demoly PPW Pirmohamed M Romano A Important questions in Allergy: 1–drug allergy/hypersensitivity Allergy Asthma Clin Immunol 2008 63 5 616 619 10.1111/j.1398-9995.2008.01693.x 18394136

[B105] Brockow KRA Blanca M Ring J Pichler W Demoly P General considerations for skin test procedures in the diagnosis of drug hyper-sensitivity Allergy 2002 57 45 51 11991289

[B106] Adkinson NF ED Jr Gruchalla R Haggerty H Kawabata T Sandler JD Updyke L Shear NH Wierda D Health and environmental sciences institute task force: Task force report: future research needs for the prevention and management of immune-mediated drug hypersensitivity reactions J Allergy Clin Immunol 2002 1098 3 S461 467 11897992 10.1067/mai.2002.122214

[B107] Gleeson M Immune function in sport and exercise J Appl Physiol 2007 103 2 693 699 10.1152/japplphysiol.00008.2007 17303714

[B108] Lakier Smith L Overtraining, excessive exercise, and altered immunity: is this a T helper-1 versus T helper-2 lymphocyte response? Sports Med 2003 33 5 347 364 10.2165/00007256-200333050-00002 12696983

[B109] Fitch KD An overview of asthma and airway hyper-responsiveness in Olympic athletes Br J Sports Med 2012 46 6 413 416 10.1136/bjsports-2011-090814 22228581

[B110] Bonini M Lapucci G Petrelli G Todaro A Pamich T Rasi G Bonini S Predictive value of allergy and pulmonary function tests for the diagnosis of asthma in elite athletes Allergy 2007 62 10 1166 1170 10.1111/j.1398-9995.2007.01503.x 17845586

[B111] Schwartz LB Delgado L Craig T Bonini S Carlsen KH Casale TB Del Giacco S Drobnic F van Wijk RG Ferrer M Exercise-induced hypersensitivity syndromes in recreational and competitive athletes: a PRACTALL consensus report (what the general practitioner should know about sports and allergy) Allergy 2008 63 8 953 961 10.1111/j.1398-9995.2008.01802.x 18691297

[B112] Jaakkola MS Jaakkola JJ Assessment of public health impact of work-related asthma BMC Med Res Methodol 2012 12 22 10.1186/1471-2288-12-22 22390159 PMC3339512

[B113] Henneberger PK Mirabelli MC Kogevinas M Anto JM Plana E Dahlman-Hoglund A Jarvis DL Kromhout H Lillienberg L Norback D The occupational contribution to severe exacerbation of asthma Eur Respir J 2010 36 4 743 750 10.1183/09031936.00135109 20351033

[B114] Kogevinas M Zock JP Jarvis D Kromhout H Lillienberg L Plana E Radon K Toren K Alliksoo A Benke G Exposure to substances in the workplace and new-onset asthma: an international prospective population-based study (ECRHS-II) Lancet 2007 370 9584 336 341 10.1016/S0140-6736(07)61164-7 17662882

[B115] Vandenplas O Lantin AC D’Alpaos V Larbanois A Hoet P Vandeweerdt M Thimpont J Speybroeck N Time trends in occupational asthma in Belgium Respir Med 2011 105 9 1364 1372 10.1016/j.rmed.2011.05.002 21624825

[B116] Paris C Ngatchou-Wandji J Luc A McNamee R Bensefa-Colas L Larabi L Telle-Lamberton M Herin F Bergeret A Bonneterre V Work-related asthma in France: recent trends for the period 2001–2009 Occup Environ Med 2012 69 6 391 397 10.1136/oemed-2011-100487 22383588

[B117] Moscato G Pala G Barnig C De Blay F Del Giacco SR Folletti I Heffler E Maestrelli P Pauli G Perfetti L EAACI consensus statement for investigation of work-related asthma in non-specialized centres Allergy 2012 67 4 491 501 10.1111/j.1398-9995.2011.02784.x 22257175

[B118] Moscato G Vandenplas O Van Wijk RG Malo JL Perfetti L Quirce S Walusiak J Castano R Pala G Gautrin D EAACI position paper on occupational rhinitis Respir Res 2009 3 10 16 19257881 10.1186/1465-9921-10-16 PMC2654869

[B119] Moscato G Pala G Boillat MA Folletti I Gerth van Wijk R Olgiati-Des Gouttes D Perfetti L Quirce S Siracusa A Walusiak-Skorupa J EAACI position paper: prevention of work-related respiratory allergies among pre-apprentices or apprentices and young workers Allergy 2011 66 9 1164 1173 10.1111/j.1398-9995.2011.02615.x 21557751

[B120] Cowl CT Occupational asthma: review of assessment, treatment, and compensation Chest 2011 139 3 674 681 10.1378/chest.10-0079 21362654

[B121] Tarlo SM Liss GM Prevention of occupational asthma Curr Allergy Asthma Rep 2010 10 4 278 286 10.1007/s11882-010-0118-y 20424999

[B122] Baur X Sigsgaard T The new guidelines for management of work-related asthma Eur Respir J 2012 39 3 518 519 10.1183/09031936.00096211 22379145

[B123] de Groene GJ Pal TM Beach J Tarlo SM Spreeuwers D Frings-Dresen MH Mattioli S Verbeek JH Workplace interventions for treatment of occupational asthma Cochrane Database Syst Rev 2011 11 5 10.1002/14651858.CD006308.pub3 21563151

[B124] Vandenplas O Dressel H Wilken D Jamart J Heederik D Maestrelli P Sigsgaard T Henneberger P Baur X Management of occupational asthma: cessation or reduction of exposure? A systematic review of available evidence Eur Respir J 2011 38 4 804 811 10.1183/09031936.00177510 21436354

[B125] Lack G Epidemiologic risks for food allergy J Allergy Clin Immunol 2008 121 6 1331 1336 10.1016/j.jaci.2008.04.032 18539191

[B126] Martinez FD New insights into the natural history of asthma: primary prevention on the horizon J Allergy Clin Immunol 2011 128 5 939 945 10.1016/j.jaci.2011.09.020 22036094 PMC3963825

[B127] DunnGalvin A Daly D Cullinane C Stenke E Keeton D Erlewyn-Lajeunesse M Roberts GC Lucas J Hourihane JO Highly accurate prediction of food challenge outcome using routinely available clinical data J Allergy Clin Immunol 2011 127 3 633 639 10.1016/j.jaci.2010.12.004 21377032

[B128] Papadopoulos NG Arakawa H Carlsen KH Custovic A Gern J Lemanske R Le Souef P Makela M Roberts G Wong G International consensus on (ICON) pediatric asthma Allergy 2012 67 8 976 997 10.1111/j.1398-9995.2012.02865.x 22702533 PMC4442800

[B129] Bozzetto S Carraro S Giordano G Boner A Baraldi E Asthma, allergy and respiratory infections: the vitamin D hypothesis Allergy 2012 67 1 10 17 10.1111/j.1398-9995.2011.02711.x 21933195

[B130] Roberts G Patel N Levi-Schaffer F Habibi P Lack G Food allergy as a risk factor for life-threatening asthma in childhood: a case-controlled study J Allergy Clin Immunol 2003 112 1 168 174 10.1067/mai.2003.1569 12847494

[B131] Auffray C Adcock IM Chung KF Djukanovic R Pison C Sterk PJ An integrative systems biology approach to understanding pulmonary diseases Chest 2010 137 6 1410 1416 10.1378/chest.09-1850 20525651

[B132] Niggemann B Jacobsen L Dreborg S Ferdousi HA Halken S Host A Koivikko A Koller D Norberg LA Urbanek R Five-year follow-up on the PAT study: specific immunotherapy and long-term prevention of asthma in children Allergy 2006 61 7 855 859 10.1111/j.1398-9995.2006.01068.x 16792584

[B133] Edgecombe K Latter S Peters S Roberts G Health experiences of adolescents with uncontrolled severe asthma Arch Dis Child 2010 95 12 985 991 10.1136/adc.2009.171579 20675501

[B134] Monks H Gowland MH MacKenzie H Erlewyn-Lajeunesse M King R Lucas JS Roberts G How do teenagers manage their food allergies? Clin Exp Allergy 2010 40 10 1533 1540 10.1111/j.1365-2222.2010.03586.x 20682004

[B135] EAACI A European Declaration of Immunotherapy. Designing the future of allergen specific immunotherapy Clinical and translational allergy 2012 2 20 10.1186/2045-7022-2-20 23110958 PMC3514324

[B136] Bilo BM Bonifazi F Hymenoptera venom immunotherapy Immunotherapy 2011 3 2 229 246 10.2217/imt.10.88 21322761

[B137] Warrington R Immunotherapy in asthma Immunotherapy 2010 2 5 711 725 10.2217/imt.10.47 20874654

[B138] Calderon M Cardona V Demoly P One hundred years of allergen immunotherapy European Academy of Allergy and Clinical Immunology celebration: review of unanswered questions Allergy 2012 67 4 462 476 10.1111/j.1398-9995.2012.02785.x 22309435

[B139] Jutel M Akdis CA Immunological mechanisms of allergen-specific immunotherapy Allergy 2011 66 6 725 732 10.1111/j.1398-9995.2011.02589.x 21466562

[B140] Fujita H Soyka MB Akdis M Akdis CA Mechanisms of allergen-specific immunotherapy Clin Transl Allergy 2012 2 1 2 10.1186/2045-7022-2-2 22409879 PMC3395833

[B141] Palomares O Rückert B Jartti T Kücüksezer UC Puhakka T Gomez E Fahrner HB Speiser A Jung A Kwok WW Induction and maintenance of allergen-specific FOXP3+ Treg cells in human tonsils as potential first-line organs of oral tolerance J Allergy Clin Immunol 2012 129 510 520 10.1016/j.jaci.2011.09.031 22051696

